# Transcriptome Sequencing and Biochemical Analysis of Perianths and Coronas Reveal Flower Color Formation in *Narcissus pseudonarcissus*

**DOI:** 10.3390/ijms19124006

**Published:** 2018-12-12

**Authors:** Xi Li, Dongqin Tang, Hui Du, Yimin Shi

**Affiliations:** 1 School of Agriculture and Biology, Shanghai Jiao Tong University, Shanghai 200240, China; lixi1324@sjtu.edu.cn (X.L.); duhui1122@sjtu.edu.cn (H.D.); 2 School of Design, Shanghai Jiao Tong University, Shanghai 200240, China; dqtang@sjtu.edu.cn

**Keywords:** *Narcissus pseudonarcissus*, transcriptome, biochemical analysis, carotenoids, CCD4

## Abstract

*Narcissus pseudonarcissus* is an important bulbous plant with white or yellow perianths and light yellow to orange-red coronas, but little is known regarding the biochemical and molecular basis related to flower color polymorphisms. To investigate the mechanism of color formation, RNA-Seq of flower of two widely cultured cultivars (‘Slim Whitman’ and ‘Pinza’) with different flower color was performed. A total of 84,463 unigenes were generated from the perianths and coronas. By parallel metabolomic and transcriptomic analyses, we provide an overview of carotenoid biosynthesis, degradation, and accumulation in *N. pseudonarcissus*. The results showed that the content of carotenoids in the corona was higher than that in the perianth in both cultivars. Accordingly, phytoene synthase (PSY) transcripts have a higher abundance in the coronas than that in perianths. While the expression levels of carotenoid biosynthetic genes, like *GGPPS*, *PSY*, and *LCY-e*, were not significantly different between two cultivars. In contrast, the carotenoid degradation gene *NpCCD4* was highly expressed in white-perianth cultivars, but was hardly detected in yellow-perianth cultivars. Silencing of *NpCCD4* resulted in a significant increase in carotenoid accumulation, especially in all-*trans*-β-carotene. Therefore, we presume that *NpCCD4* is a crucial factor that causes the low carotenoid content and color fading phenomenon of ‘Slim Whitman’ by mediating carotenoid turnover. Our findings provide mass RNA-seq data and new insights into carotenoid metabolism in *N. pseudonarcissus*.

## 1. Introduction

*Narcissus pseudonarcissus*, also known as daffodil, is an ornamental bulbous plant in the Amaryllidaceae family. *N. pseudonarcissus* is popular in the world flower market because of its unique flower shape, flower color, and sweet fragrance [1,2,3,4]. Flower color is one of the most important ornamental characters for *N. pseudonarcissus*. However, flower color was monotonous for most cultivars in this species, usually white to yellow in the perianths, and light yellow to orange-red in the coronas [1]. Previous studies have shown that component and content of carotenoids are the main factors that affected the flower color of *N. pseudonarcissus* [5,6] and a total of ten kinds of carotenoids were separated from the perianths and coronas of fifteen *N. pseudonarcissus* cultivars [7].

Carotenoids, mostly C40 terpenoids, are the second conspicuous naturally occurring pigments and they participate in multiple biological processes in plants, such as photoprotection and the regulation of growth and development [8,9]. Meanwhile, carotenoids are also indispensable for human diet as antioxidants and precursors of provitamin A [10]. Due to their importance, the carotenoids biosynthesis pathway has been extensively characterized in numerous plants in the past decades [8,11]. As a carotenoid-enriched horticultural crop, *N. pseudonarcissus* played an important role in 1990s in the process of cloning genes encoding important enzymes, such as phytoene synthase (PSY) and phytoenede saturase(PDS) [12]. Subsequently, cDNA coding for PSY from *N. pseudonarcissus* was transformed into rice, which was the first transgenic attempt for accumulation of carotenoids in a carotenoid-lacking plant [13]. However, mechanisms that control carotenoids metabolize in cultivars of *N. pseudonarcissus* with different colors are far from conclusive. Nevertheless, carotenoid metabolism is a complicated process that the upstream metabolites could turn into downstream carotenoids by a series of desaturation and isomerization and feedback mechanism coordinates were exiting to ensure the proper production of various carotenoids. Meanwhile, carotenoid accumulation is dependent on the dynamic metabolic equilibrium between biosynthesis and degradation [8,11]. Carotenoid cleavage deoxygenases (CCDs), which degrades β-carotene and downstream carotenoids and produces apocarotenoids, abscisic acid(ABA), strigolactones, and other volatile compounds [14], are the main cause of carotenoids biodegradation in many horticultural plants, such as chrysanthemums [15], peach [16,17], and *Crocus* [18]. Nine CCD genes have been identified in the *Arabidopsis* genome, including *AtCCD1*, *AtCCD4*, *AtCCD7*, *AtCCD8*, *AtNCED2*, *AtNCED3*, *AtNCED5*, *AtNCED6*, and *AtNCED9* [19]. In horticultural plants, CCDs are usually named according to their sequence similarity to the *Arabidopsis* CCDs. However, no CCD genes have been declared in *Narcissus* up to now.

With the increase of usability and efficiency of RNA sequencing (RNA-Seq) tools, the study of gene models for metabolic pathways underlying metabolite variation has been promoted, especially in non-model plants, such as flavonoid biosynthesis studies in yam [20], freesia [21], and strawberry [22], and anthocyanins accumulation analysis in *Paeonia* [23]. However, without metabolomics, only a partial understanding can be achieved from transcriptome alone, because not all genes that are transcribed could translate into functional enzymes and temporal scales of translation and metabolite formation may strongly diverge [24]. Metabolomics, which are closer to the plants’ phenotypes, has developed into a powerful approach to understand the accumulation of specific metabolites. The biochemical results that were observed by HPLC-MS/MS or GC-MS, such as anthocyanin pigments and flavonoid intermediates in flower petal [25,26] and phosphorylated metabolites in oat root [27], could be interpreted in light of the expression levels of related genes from trancriptome analysis. Therefore, it is more likely to have an insight into the metabolic flow by a comprehensive approach, not only to study the transcriptional level, but also to compare the related metabolites.

In the present study, the first RNA-Seq project for the perianth and corona tissues of *N. pseudonarcissus* was performed while using the Illumina sequencing technique. The perianth and corona samples that were used for RNA-seq were collected from two widely cultivated *N. pseudonarcissus* cultivars, ‘Slim Whitman’ and ‘Pinza’. Through an integrative approach that combined biochemical analysis with bioinformatics, the carotenoids metabolic pathways that are related to the flower color formation of *N. pseudonarcissus* were illuminated and the essential genes responsible for color formation were examined.

## 2. Results

### 2.1. Flower Phenotype and Quantitation of Carotenoid Contents in ‘Slim Whitman’ and ‘Pinza’

**Figure 1:**
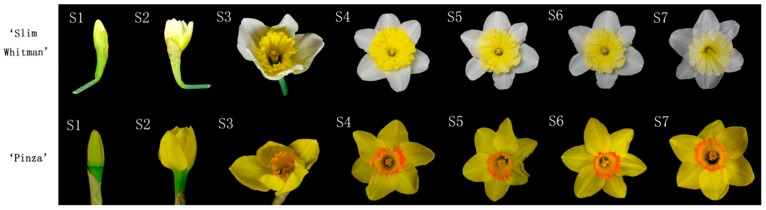
Phenotypic characters of flowers in different stages. S1–S4: from flower bud to blooming period. S4–S7: blooming to fading. The upper panel is cultivar ‘Slim Whitman’, S1, flower unopened, the corona was not pigmented yet; S2 and S3, flower was opening, white perianth and yellow corona were observed; at the blooming stage (S4), as the flower fully opened, the corona showed to be bright yellow; after that, the corona color began to fade (S5 to S7). Under natural conditions, about 15 days after blooming, the corona gradually fading to pale yellow or even white. The lower panel is cultivar ’Pinza’ in different developmental stages. ‘Pinza’ has yellow perianth and yellow-orange corona. At stage 3 (S3), the corona gradually obtained an orange cycle at the rim, which greatly enhanced its ornamental value. The yellow perianth and corona with orange cycle did not show any depigmentation and remained vivid until the perianth began to wrinkle in S6 and S7. The perianths and coronas in stage S4 were used to carry out the following RNA-Seq experiments, and the perianths and coronas in stage S2–S7 were used to carry out metabolome-related experiments, respectively.

The ‘Slim Whitman’ (SW) cultivar had white perianth and light yellow corona. The corona of SW gradually faded to pale yellow or even white during S5 to S7 (Figure 1). ‘Pinza’ (PZ) has yellow perianth and yellow-orange corona. Unlike that of SW, the perianth and corona of PZ did not show any depigmentation during the whole process of flowering.

**Figure 2:**
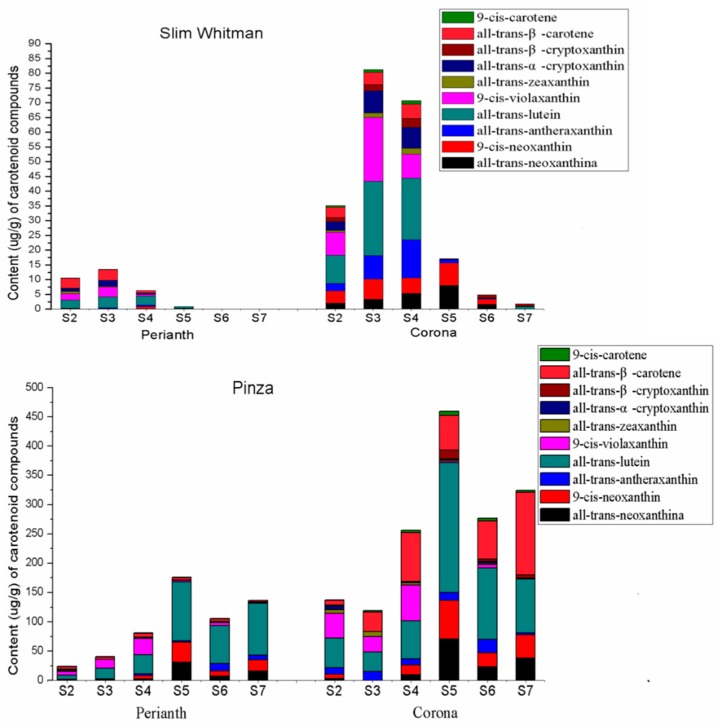
The content (μg/g) of carotenoid compounds in perianths and coronas of SW and PZ in different stages. The carotenoids are all-*trans*-neoxanthin, 9-*cis*-neoxanthin, all-*trans*-antheraxanthin, all-*trans*-lutein, 9-*cis*-violaxanthin, all-*trans*-zeaxanthin, all-*trans*-α-cryptoxanthin, all-*trans*-β-cryptoxanthin, all-*trans*-β-carotene, and 9-*cis*-β-carotene, respectively.

To understand the color divergence at the biochemical level, the metabolomic profiles of carotenoids that accumulated in perianths and coronas of SW and PZ from S2 to S7 were compared using HPLC-MS/MS (Figure 2 and Supplementary Table S1). A total of 10 carotenoids were detected in all of the samples, including all-*trans*-neoxanthin, 9-*cis*-neoxanthin, all-*trans*-antheraxanthin, all-*trans*-lutein, 9-*cis*-violaxanthin, all-*trans*-zeaxanthin, all-*trans*-α-cryptoxanthin, all-*trans*-β-cryptoxanthin, all-*trans*-β-carotene, and 9-*cis*-β-carotene (Supplementary Figure S1).

Consistent with the visual inspection, total carotenoid (TC) contents in the coronas were higher than those in the perianths in any developmental stages of both SW and PZ (Figure 2 and Supplementary Table S1). In the coronas of SW (SWC), the TC was increased at first and then decreased, which peaked at S3 (81.176 μg/g). Dramatic reductions in carotenoid accumulation were detected after S4, which were 17.128, 4.822, and 1.751 μg/g, at S5, S6, and S7, respectively. A similar trend was observed in perianth of SW (SWP). TC in SWP from S2 to S4 were 10.610, 13.573, and 6.381 μg/g, respectively, followed a sharp decrease after S4, and only 0.830 μg/g TC was detected at S5 and was barely detected at S6 and S7. In contrast, in the coronas of PZ (PZC), the carotenoid content was remarkably higher (about four times of SWC at S2 and S4; 27 times of SWC at S5; 57 times of SWC at S6) and did not decrease significantly at the late flowering stages. In the perianth of PZ (PZP), the TC contents from S2 to S7 were maintained at a relatively high level, and they peaked at S5 (175.852 μg/g).

In SWP, all-*trans*-lutein, 9-*cis*-violaxanthin, and all-*trans*-β-carotene turned to be the major components. In PZP, the all-*trans*-lutein was the most dominant pigment, accounting for 46.0%, 40.1%, 57.6%, 61.9%, and 64.7% of TC at S3, S4, S5, S6, and S7, respectively. Meanwhile, the 9-*cis*-violaxanthin and all-*trans*-β-carotene were also relatively abundant in PZP. Unlike that in the perianth, in SWC, the all-*trans*-lutein and 9-*cis*-violaxanthin accounted for the majority from S2 to S4, while along with the reduction of other carotenoids, all-*trans*-neoxanthin and 9-*cis*-neoxanthin became the principal components at S5 and S6. In PZC, all-*trans*-lutein and 9-*cis*-violaxanthin were the dominant components at S2, but all-*trans*-β-carotene began to accumulate in large quantities from S3, and taking up to 27.6%, 32.5%, 12.8%, 23.5%, and 43.6% at S3, S4, S5, S6, and S7, respectively. When carotenoid amounts of SWC and PZC were compared, the most significant difference was on all-*trans*-β-carotene, which was as high as 83.369 μg/g in PZC and only 4.680 μg/g in SWC at S4. Moreover, the all-*trans*-β-carotene was not detected from S5 to S7 in SWC, while possessing a very high content in PZC. Taking into account the fact that all-*trans*-β-carotene was the main orange pigment among the ten detected carotenoids, we assume it was the decisive factor that is attributed to the orange rim on the corona of PZ. In general, the variation tendency of carotenoid content was consistent with the flower color phenotypic variation, as described above.

We further assessed components and contents of carotenoids of 27 hybrids of ‘Slim Whitman’ and ‘Pinza’ at S4 (Supplementary Table S2). The results demonstrated that the white perianths hybrids contained less carotenoids, with 0.65 to 7.85 μg/g TC in the perianths. While, the TC contents in the yellow perianths ranged from 16.96 to 82.88 μg/g, among which all-*trans*-neoxanthin, all-*trans*-lutein and 9-*cis*-violaxanthin were the dominant components. Among the 27 hybrids, we obtained four with orange rims on the coronas, and all of them were rich in all-*trans*-β-carotene as compared with the others. It is obvious that the content of carotenoids, especially all-*trans*-β-carotene, is positively correlated with flower color. However, in order to fully understand the carotenoid metabolism, it is necessary to detect the gene expression at the transcriptional level.

### 2.2. RNA-Seq and Functional Annotation

To elucidate the mechanism of flower color formation and carotenoid metabolism, we performed RNA-seq for the SWP, SWC, PZP, and PZC at S4. In total, approximately 67.13 Gb Clean Data was obtained from the eight samples (SWP-1, SWP-2; SWC-1, SWC-2; PZP-1, PZP-2; PZC-1, PZC-2). After the removal of the adapter sequences and low quality sequences, approximately 26.2, 26.5; 35.1, 23.9; 34.4, 25.5; 30.1, 25.6 million clean reads were left for the SWP, SWC, PZP, and PZC transcriptomes, with GC percentages of 49.46%, 49.19%; 49.52%, 49.66%; 49.39%, 48.87%, 45.93%, and 45.94%, respectively. The accession numbers of all raw data in the Short Read Archive (SRA) Sequence Data base in the National Center for Biotechnology Information (NCBI) were listed in Supplementary Table S3.

**Figure 3:**
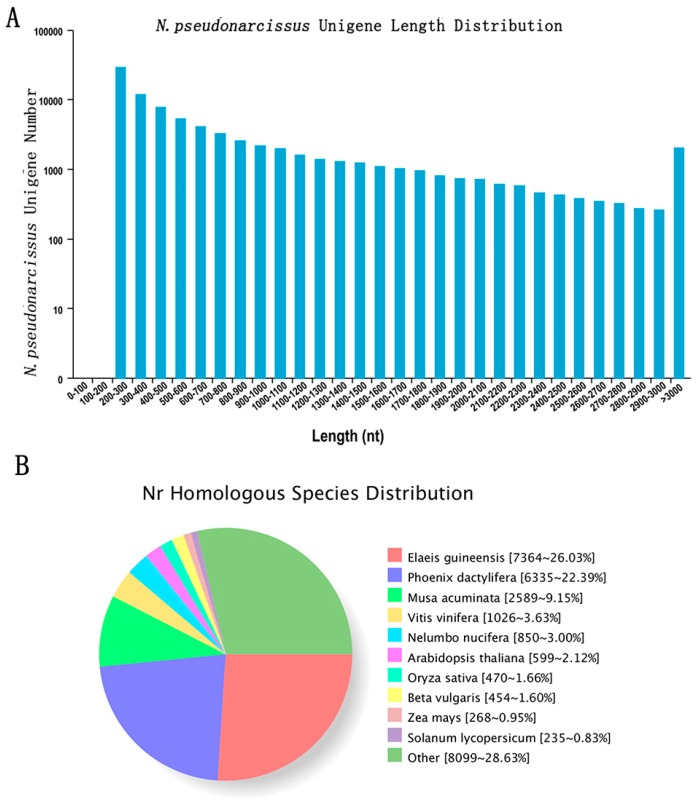
The *N. pseudonarcissus* sequence length distribution and Nr homologous species distribution. (**A**): The sequence length distribution of assembled unigenes. The horizontal axis represents the sequence length of base pairs and vertical axis represents the number of assembled unigenes in the range of the sequence length. (**B**): Species distribution of the top BLAST hits.

All clean reads from the eight libraries were subsequently de novo assembled by the Trinity program and a total of 84,463 unigenes with an average length of 741.72 bp, and 50% of the assembled bases were incorporated into unigenes of 1239 bp or longer. The size distribution of the *N. pseudonarcissus* unigenes is given in Figure 3A, with 21.98% of all unigenes showing lengths longer than 1 kb.

Gene function was annotated based on the following databases: NR, Pfam, KOG/COG/eggNOG, Swiss-Prot, KEGG and GO. A total of 30,156 unigenes (36.88%) were annotated based on the public protein databases. A total of 28,377 unigenes (92.8% of assembled unigenes) had a match in the NR database, and 9164 (29.98%), 16,065 (52.56%), 9874 (32.30%) 16,309 (53.35%), 19,438 (63.59%), 17,257 (56.46%), and 27,795 (90.93%) unigenes showed significant similarity to sequences in the COG, GO, KEGG, KOG, Pfam, Swiss-Prot, and eggNOG databases, respectively (Table 1). Among the 28,377 unigenes that were matched in the NR database, 7364 unigenes had closest matches with sequences of *Elaeis guineensis*, followed by *Phoenix dactylifera* (6335), *Musa acuminate* (2589), *Vitis vinifera* (1026), and *Nelumbo nucifera* (850) (Figure 3B).

### 2.3. Differential Expression Analysis of Assembled N. pseudonarcissus Unigenes

Differential expression analysis was performed between SWP and SWC, SWP and PZP, SWC and PZC, and PZP and PZC of *N. pseudonarcissus*. Totally, we found 730 differentially expressed unigenes (DEGs) (352 up-regulated and 378 down-regulated) between SWP and SWC, 1022 (511 up-regulated and 511 down-regulated) between SWP and PZP, and 3880 (2232 up-regulated and 1648 down-regulated) between SWC and PZC, 3317 between PZP and PZC (1636 up-regulated and 1681 down-regulated), respectively (Table 2). Volcano plots were constructed to describe the distribution of significant differentially expressed genes (Supplementary Figure S2). Functional annotation of the identified DEGs is carried out, and the number of genes annotated by different databases is listed in Supplementary Table S4. In order to further understand of the biological functions of the DEGs, they were annotated with GO (Supplementary Figure S3) and COG (Supplementary Figure S4).

DEGs enrichment analyses that are based on KEGG pathways were subsequently performed. When all of the DEGs were checked against the KEGG pathways database, the DEGs between SWP and SWC, SWP and PZP, SWC and PZC, PZP and PZC were linked to 124, 92, 442, 490 KEGG pathways, respectively. In the KEGG enrichment analysis, the first 20 pathways with the most significant and reliable Q value were selected to display in the supplementary material (Supplementary Figure S5). DEGs were highly enriched in the metabolism pathway “Carotenoid biosynthesis” (ko00906) in the comparison SWP and PZP, SWC and PZC, and PZP and PZC, with the enrichment factor as 3.91, 1.90, and 2.45, respectively. Meanwhile, the DEGs enrichment factor of “Carotenoid biosynthesis” (ko00906) in SWP and SWC was also as high as 2.90, indicating that the gene expression profile in the carotenoid biosynthesis pathway had obvious differences among the samples. In addition, “Monoterpenoid biosynthesis” (ko00902) was one of the most enriched pathways when comparing the DEGs between SWP vs. SWC and PZP vs. PZC, possessing extraordinary enrichment factors as 30.95 and 7.83. “Degradation of aromatic compounds” (ko01220) was also significant between SWP and PZP, with an enrichment factor of 6.95.

### 2.4. Comparison of Transcriptional Profiles of Genes Involved in Carotenoid Biosynthesis Pathway and Validation by Quantitative Real-Time PCR (qRT-PCR)

**Figure 4:**
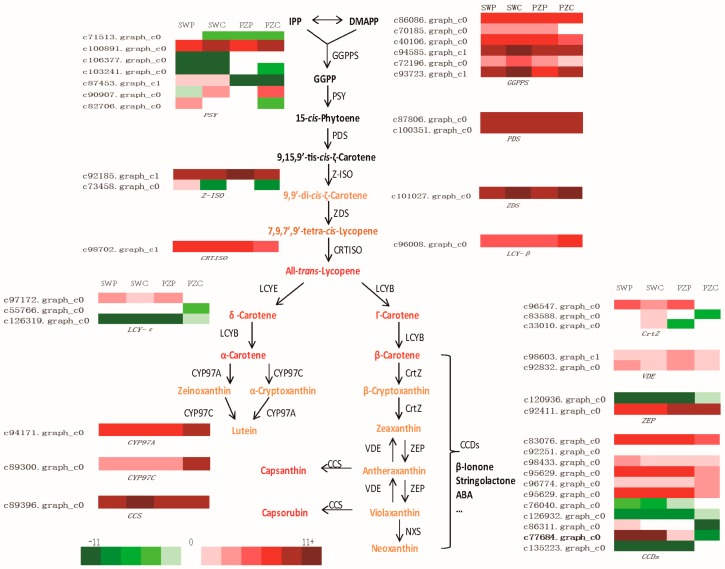
The carotenoid biosynthesis process with its core metabolites and enzymes and the expression levels of core enzyme genes. Enzyme names and expression patterns are indicated at the side of each step. Metabolites are bolded and colored according to their compound colors, and black indicates no color. Color boxes from left to right represent unigenes showing lower or higher expression level in perianth of Slim Whitman (SWP), coronas of Slim Whitman (SWC), perianth of ‘Pinza’ (PZP), and corona of ‘Pinza’ (PZC), respectively. Color saturation represents magnitude of log_2_ (expression ratio) for each unigenes, with the FPKM values 0–0.0019, 0.0019–0.008, 0.008–0.03125, 0.03125–0.125, 0.125–0.5, 0.5–2, 2–8, 8–32, 32–128, 128–512, 512–2048, and ≥2048 represented by scale levels −11 to 11, respectively.

Core metabolites and genes in the “Carotenoid biosynthesis” pathway were studied in detail to find the key transcripts that influence the accumulation of carotenoids (Figure 4). Carotenoid biosynthesis started with GGPPS (GGPP synthase) to synthesize GGPP from isopentenyl diphosphate (IPP) and dimethylallyl diphosphate (DMAPP). There were six unigenes annotated as *GGPPS*, among which c94585.graph_c1 had a relatively higher expression in the coronas (FPKM as 4156.34 and 913.15 in SWC and PZC, respectively) than that in the perianth (FPKM as 805.91 and 894.24 in SWP and PZP, respectively). Subsequently, two GGPP molecules were condensed into colorless phytoene by PSY (phytoene synthase) and a total of seven unigenes were annotated as *PSY*, among which c100891.graph_c0 was the dominant one and expressed obviously higher in the coronas (FPKM as 709.42 and 465.04) than those in the perianths (FPKM as 94.52 and 112.04) of SW and PZ. PDS (phytoenede saturase) and ZDS (z-carotene desaturase) were two important phylogenetically related enzymes that catalyzed symmetric dehydrogenation reactions converting 15-*cis*-phytoene to tetra-*cis*-lycopene. Unigenes annotated as *PDS* showed no significant divergence in different cultivars or tissues, while the expression levels of *ZDS* were higher in the coronas than that in the perianths. Unigene, annotated as *CRTISO* (carotenoid isomerase), which converted tetra-*cis*-lycopene to the red all-*trans*-lycopene had no palpable expression difference among the four samples. *β*-carotene and its derivatives relied on the cyclization of lycopene generated by LCY-β (lycopene b-cyclase). The unigene annotated as LCY-β c96008.graph_c0 had a higher expression level in PZC, about three-fold of SWP, SWC, and PZP. LCY-ε (Lycopene ε-cyclase, c97172.graph_c0) showed a higher expression in the perianths than that in the coronas, both in SW and PZ. As a contrast, the amount of expression of 94171.graph_c0 (CYP97A) in the coronas was twice of that in the perianths. Afterwards, β-carotene was further hydroxylated by CrtZ to produce cryptoxanthin and zeaxanthin, which were also among the carotenoid pigments in the perianths and coronas of *N.pseudonarcissus*. The FPKM values of *CrtZ*-like unigenes were not high in all samples, and there was no significant difference. Zeaxanthin was hydroxylated by ZEP (zepoxidase) to yield antheraxanthin and then violaxanthin. The unigenes of *ZEP*-like sequences showed a slightly higher expression level in PZ than that in SW. By transcriptional comparison of the carotenoid synthesis genes, we found that the expression of *PSY*, which had obvious difference between perianths and coronas, was positively related to TC.

It was noteworthy that the unigene c77684.graph_c0 (CCD) showed a significantly higher level in SW than in PZ (Figure 4), more than 500 times higher in the perianths and 2000 times higher in the coronas. The unigene c77684.graph_c0 was annotated as zeaxanthin 7, 8 (7′,8′)-cleavage dioxygenase, and/or 9-*cis*-epoxycarotenoid dioxygenase in the KEGG database, and predicted as 7, 8 (7′,8′)-cleavage dioxygenase in NR database. C77684.graph_c0 transcripts had extremely high levels of expression in SW, indicating that carotenoids were undergoing rapid degradation while they were synthesizing, which was almost non-existent in Pinza. This was a powerful explanation for that SW had less carotenoid accumulation and discolored over time.

**Figure 5:**
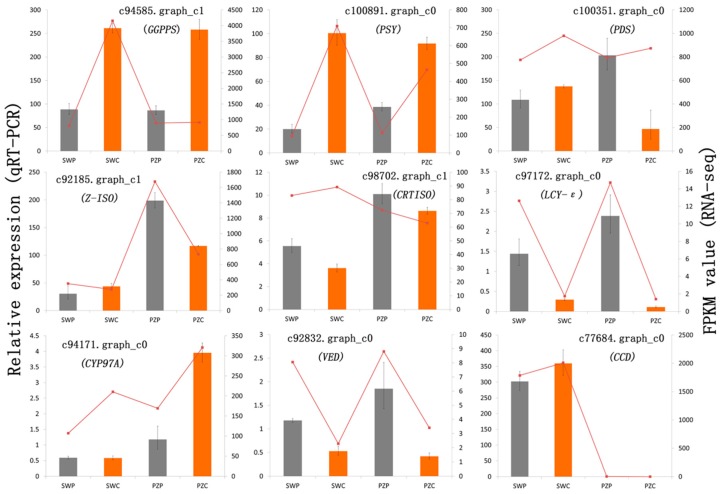
Comparison of expression profiles of nine representative genes from SWP, SWC, PZP, and PZC as measured by RNA-seq and quantitative real-time PCR (qRT-PCR). The nine genes are assigned to the of carotenoid biosynthesis pathway in Figure 4. Columns represent expression determined by qRT-PCR (left y-axis), while lines represent expression by RNA-seq in FPKM values (right y-axis). The x-axis in each chart represents the SWP, SWC, PZP and PZC, respectively. For qRT-PCR assay, the mean was calculated from three biological replicates each with three technical replicates (*n* = 9). For RNA-seq, each point is the mean of two biological replicates. Correlations between qRT-PCR and RNA-seq expressions were calculated and their associated *p*-values are 7.53 × 10^−10^. R^2^ = 0.823.

To verify the expression of DEGs that were identified from the transcriptome sequencing in the carotenoid biosynthesis pathway, qRT-PCR analyses were performed using SWP, SWC, PZP, and PZC at S4. Nine unigenes were selected for validation by qRT-PCR and their relative expression showed a similar trend with the transcriptome sequencing (Figure 5). There was a significant positive correlation between relative expression by qRT-PCR and the FPKM value, with a relatively high correlation coefficient (R^2^ = 0.823).

**Figure 6:**
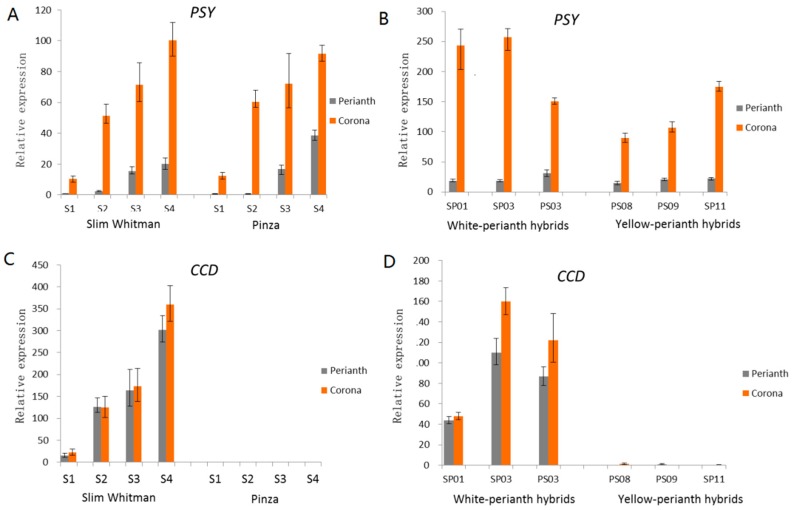
The expression pattern of unigene c100891.graph_c0 (*PSY*) and c77684.graph_c0 (Carotenoid cleavage deoxygenases (*CCD*)) by qRT-PCR. The Actin was used as an internal control for normalization. Total RNA was extracted from the perianth and corona at different flowering stages. (**A**) The expression pattern of *PSY* in perianths and coronas of SW and PZ from S1 to S4. (**B**) The expression pattern of *PSY* in perianths and coronas during S4 of six hybrids of SW and PZ. (**C**) The expression pattern of *CCD* in perianths and coronas of SW and PZ from S1 to S4. (**D**) The expression pattern of *CCD* in perianths and coronas during S4 of six hybrids of SW and PZ. SP01, SP03, and PS03 were white-perianth hybrids; PS08, PS09, and SP11 were yellow-perianth hybrids.

Based on the gene expression patterns of the carotenoid metabolic pathways, we concluded that upstream genes, such as *GGPPS* and *PSY*, had a higher expression in coronas than that in perianths. In many cases, PSY was considered to be a rate-limiting enzyme in the carotenoid biosynthesis and it catalysed the biosynthesis of 15-cisphytoene, the primary precursor for all classes of carotenoid [2,28]. The expression level of *PSY* in the coronas was higher than that in perianths from S1 to S4 in both SW and PZ (Figure 6A), which may interpret the fact that the total amount of carotenoids in the coronas was always more than that in the perianths in both SW and PZ. However, there was no obvious difference on the expression of *PSY* between SW and PZ. The expression pattern of *PSY* in hybrids of SW and PZ was further examined and the results showed that the expression of *PSY* in the coronas was higher than that in the perianths in all the hybrids, but there is no significant difference between yellow-perianth and white-perianth hybrids (Figure 6B). Therefore, we speculate that *PSY* only affects the carotenoid accumulation between perianth and corona within the individual cultivar, but it is not the key factor that causes different color phenotype in different cultivars.

On the other hand, the most significant divergence between the two cultivars reflected on the *CCD* (c77684.graph_c0), which had high expression levels in SWP and SWC (FPKM values were 1786.39 and 2008.92, respectively), but were barely expressed in PZP and PZC (FPKM values are 3.265 and 0.00, respectively). The expression patterns of *CCD* (c77684.graph_c0) transcripts in SW and PZ were analyzed (Figure 6C). Its expression in the perianth and corona of SW remained high and increased gradually from S1 to S4 and peaked at S4 (Supplementary Figure S6). However, in PZ, the c77684.graph_c0 had extremely low expression level in both the perianth and corona during the whole process of flowering. In the six hybrids, SP01, SP03, and PS03 possessed white perianths and had low carotenoids contents, correspondingly, the expression level of *CCD* were relatively high in these hybrids. However, *CCD* was almost not expressed in the yellow-perianth hybrids (PS08, PS09, and SP11), like that in PZ (Figure 6D). As a carotenoid cleavage dioxygenase gene, CCD plays a critical role in the carotenoid metabolism pathway and could degrade β-carotene and other downstream carotenoids [14,28]. It was considered that the C40 carotenoids in the perianth and corona of SW and white-perianth hybrids were undergoing degradation by CCD as soon as they were generated, which was a reasonable explanation for the lack of carotenoids and color fading phenomenon in SW. On the contrary, carotenoids in PZ and yellow-perianth hybrids avoided being degraded by CCD and accumulated to exhibit to be yellow to orange. Thus, we concluded that unigene c77684.graph_c0 (*CCD*) was a critical gene affecting flower color in the two cultivars.

### 2.5. Characterization of NpCCD4

In horticultural plants, members of the CCD enzyme family are named according to their amino acid sequence similarity to the *Arabidopsis* CCDs. Sequence comparison revealed that the predicted amino acid sequences of unigene c77684.graph_c0 exhibited stronger similarity to AtCCD4 proteins as compared with other CCDs (Supplementary Figure S7). The sequence of c77684.graph_c0 (*NpCCD4*) was cloned and submitted to GenBank (NCBI acc. MH918680). The nucleotide sequence of *NpCCD4* contained a complete ORF of 1806 bp corresponding to 601 amino acids, and no intron was present in the genomic clone of *NpCCD4*, which was the same as *AtCCD4* [29]. Sequence analyses revealed that the NpCCD4 proteins also contained four highly conserved histidine residues, which were typical ligands of a non-haem iron cofactor required for (di)-oxygenase activity, just the same as that in AtCCD4. Conserved glutamates or aspartate that were previously described as for fixing iron-ligating histidines [30] were also found in NpCCD4 (Supplementary Figure S8). In addition, the NpCCD4 proteins contained a predicted chloroplast transient peptide in its N-terminal region (prediction algorithm: http://www.cbs.dtu.dk/services/ChloroP).

**Figure 7:**
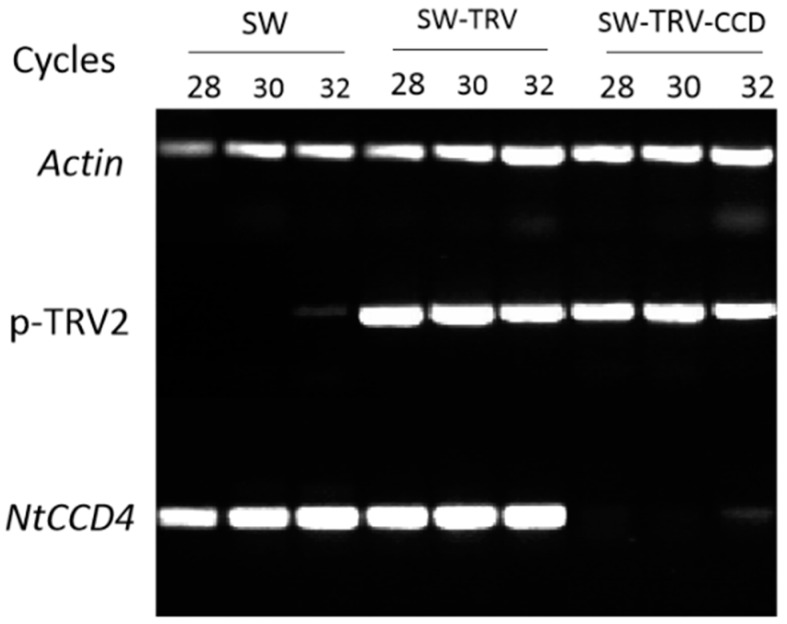
Real-time PCR (RT-PCR) analysis of *NpCCD4* expression in silenced coronas of SW by virus-induced gene silencing (VIGS). Coronas of SW during S1 were injected with *A. tumefaciens* containing TRV control (SW-TRV, pTRV1 + pTRV2), TRV carrying a *NtCCD4* fragment (SW-TRV-CCD, pTRV1 + pTRV2-NtCCD4). A total of 28, 30, and 32 PCR cycles were used for detection of *Actin*, *NpCCD4*, and pTRV2, respectively.

**Figure 8:**
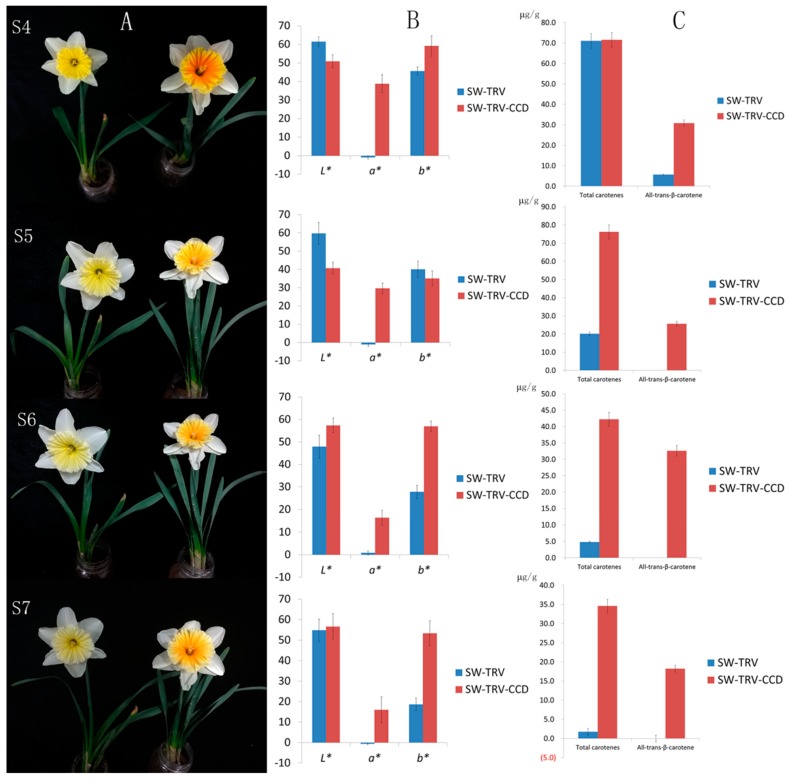
The phenotype and carotenoid contents of NtCCD4-silenced coronas during S4-S7 of SW. a, the phenotype of the tobacco rattle virus (TRV) control (left) and NtCCD4 silenced (right) plants. (**A**): Coronas of NtCCD4 silenced plants changed to be orange red, while the TRV controlled plants did not showed any difference from the wild type SW. (**B**): the chromaticity parameters L*, a* and b* of the coronas of TRV control and NtCCD4 silenced plants measured by CIE 1976 L* a* b*. In the CIE 1976 L* a* b* system, the L* value represents lightness of shade ranging from 0 (black) to 100 (white), the color parameter a* positive are for red and negative are for green, and color parameters b* positive are for yellow and negative are for blue. The higher a* value are, the color closer to red. (**C**): the total carotenoid content and all-trans-β-carotene content in coronas SW-TRV and SW-TRV-CCD (μg/g).

To determine whether *NpCCD4* encode a functional carotenoid cleavage dioxygenase, we silenced the NpCCD4 in corona of SW using a virus-induced gene silencing (VIGS) approach (Figure 7). The NpCCD4-silenced corona exhibited as orange red at S4, and it had a distinct difference from SW (Figure 8A). The chromaticity of corona was measured by CIE 1976 L* a* b* (CIELAB), among which the parameter a* was 38.84 in the corona of NpCCD4-silenced plants, while they were −0.88 and 1.65 in the SW-TRV and SW wild type. As flower gradually matured, the corona of SW-TRV control as well as SW gradually discolored and turned white, accordingly, the b* value representing the yellow degree gradually reduced. In contrast, in the CCD-silenced plants, the corona was still orange at S6 and S7, with a* value being maintained at a high level and decreased slightly at S7 (Figure 8B).

Moreover, carotenoid components and contents in SW-TRV-CCD coronas were also detected. The results showed that at S4, the TC in coronas of SW-TRV and SW-TRV-CCD, were similar, 71.05 and 71.56 μg/g, respectively. While all-*trans*-β-carotene showed a significant difference, 5.68 and 30.79 μg/g, respectively. According to this result, we considered that it was the elevated content of all-*trans*-β-carotene caused the corona color of SW-TRV-CCD to orange red. At the S5-S7 stages, due to the continuous expression of CCD, the TC decreased sharply and the all-*trans*-β-carotene decreased to 0 in coronas of SW and SW-TRV control. Nevertheless, in SW-VIGS-CCD plants, TC had a slight increase at S5, owing to the biosynthesis and a slow decline at S6 and S7. Meanwhile, the all-*trans*-β-carotene peaked at S6 and then decreased, which were basically in accordance with the observed phenotypes (Figure 8C).

Taken all together, the *NpCCD4* had the bioactivity of degrading carotenoids, and silencing of *NpCCD4* resulted in carotenoid accumulation, especially all-*trans*-β-carotene, in *N. pseudonarcissus*. The expression levels of *NpCCD4* were the decisive factor affecting the flux of various carotenoids and flower color in different cultivars.

## 3. Discussion

### 3.1. Carotenoids in N. pseudonarcissus

Carotenoids, ranging from colorless to yellow and red, are the second most abundant natural pigments on earth, such as lutein from marigold flowers [31], β-carotene from carrots, lycopene from tomatoes, and capsanthin from red peppers [8]. In this study, 10 kinds of carotenoids were detected from two *N. pseudonarcissus* cultivars, among which all-*trans*-β-carotene and 9-*cis*-β-carotene were orange because of their double bond distribution (Supplementary Figure S1). Carotenoids of interest, like β-carotene, lutein, and zeaxanthin, were all abundant in PZ and yellow-perianth hybrids (Figure 2 and Supplementary Table S1). Moreover, the existence of downstream metabolites, such as all-*trans*-neoxanthin and 9-*cis*-neoxanthin, indicated that there was a complete synthetic pathway for the biosynthesis of various carotenoids in *N. pseudonarcissus*, which were demonstrated by the transcriptional analysis. Therefore, *N. pseudonarcissus* could be used as a good experimental system for the study of carotenoid biosynthesis and metabolism.

In general, carotenoids in both SW and PZ had substantially same compositions in coronas and perianths, but with much higher concentrationin the coronas. When comparing different cultivars, we found that in SW, although the total accumulation and flux of various carotenoids were low, it did not lack any specific carotenoid, and the main components were the same as in the PZ, only with lower content. In different developmental stages, the change of carotenoids content was in cooperativity, suggesting that the contents of carotenoid components were related to each other and the various carotenoids should be studied as an organic unit (Figure 2).

### 3.2. Illumina Sequencing of N. pseudonarcissus

As a perennial bulbous plant, *N. pseudonarcissus* has a long life cycle (more than five years from sowing to anthesis) and has complex genetic background: chromosome number varies from 2*n* = 14 to 2*n* = 42 throughout the genus [32] and aneuploidy and polyploidy are quite common, most cultivars are not homozygous. Meanwhile, PCR-based molecular markers and high-density genetic maps are lacking. Thus, genetic analysis of target traits is difficult in *N. pseudonarcissus*. Under such a biological background, transcriptome analysis provides effective tools for further research on complicated traits, such as flower color.

RNA-sequencing is an efficient high-throughput approach that has been extensively used on horticultural plants, even without a reference genome, such as *Paeonia* [23] and safflower [33]. A previous high-throughput transcriptome analysis of ‘Andrews choice’ and ‘Carlton’ varieties of *N. pseudonarcissus* has been completed, but it utilized pooled RNA from frozen basal plate (SRX739241 and SRX739242). In a recent study, RNA sequencing of *N. pseudonarcissus* ‘King Alfred’ bulbs was performed to have a comprehensive understanding of the amaryllidaceae alkaloids biosynthetic pathway, and 195,347 transcripts were yielded after assembled [34]. Herein, RNA-sequencing was applied to the perianth and corona tissues of *N. pseudonarcissus* transcriptome for the first time, aiming to discover and measure DEGs that are related to flower color. In our study, we obtained 67.13 Gb clean reads, which were assembled into 84,463 unigenes using de novo assembly. These data provided a foundation for further studies on flower-related polymorphism in *N. pseudonarcissus*.

For RNA-seq to discover DEGs, reasonable sampling is the most critical step for experiment design. Different sampling methods were adopted for different research objectives [25,26,35]. In this study, we sequenced the transcriptome of two tissues (the perianths and coronas) from two cultivars with distinct flower color. Thus, in the analysis process, the four samples can be compared with each other: the same tissues from different genotypes or different tissues from the same cultivar were compared, respectively. Through this method, satisfactory results containing massive differential expression data were achieved. In total, 1022 and 3880 DEGs were identified in SWP vs. PZP and SWC vs. PZC; 730 and 3317 DEGs were identified in SWP vs. SWC and PZP vs. PZC. The DEGs were highly enriched in “Carotenoid biosynthesis” (ko00906) and other flower color related pathways, confirming that RNA-Seq combining differential expression analysis of assembled unigenes was an efficient method to dig candidate genes that are responsible for flower color polymorphism.

### 3.3. Genes Affecting Flower Color of N. pseudonarcissus

Based on the transcriptome sequencing complemented with HPLC carotenoid profiling, we have proposed the metabolic pathway of carotenoids in the perianths and coronas of *N. pseudonarcissus* (Figure 4). The differential expression analysis identified several DEGs in the four samples. Firstly, we focus on PSY, the rate-limiting enzyme in carotenoid biosynthesis in different plant species [36,37]. If PSY reactions were strongly constrained, nearly all kinds of carotenoids would be effectively eliminated. Over-expression of PSY would conduct to enhance of total carotenoid contents in *Brassica napus* and potato [38,39]. In our study, the expression of PSY was substantially higher in the coronas than that in the perianths in both SW and PZ from S1 to S4, but it was not significantly different among cultivars and hybrids. This was consistent with the TC in the coronas being always higher than that in the perianths, indicating that PSY was mainly related to the divergence of carotenoid accumulation in perianths and coronas for the same cultivar. In addition, transcriptional levels of downstream carotenoid biosynthetic genes in the four samples were similar, but the TC varied notably in different cultivars, suggesting that there were other ways of regulating the accumulation of carotenoids in different cultivars.

The total accumulation of carotenoids is a metabolic equilibrium of biosynthesis, degradation, and stable storage. The cleavage activity of CCDs, which leads to the degradation of C40 carotenoids into apocarotenoids, is pivotal in regulating carotenoid accumulation. Overexpression of *CCD4* in *Chrysanthemum* resulted in loss of carotenoids and color of petal turned from yellow to white [15]. In peach, CCD4 was the main factor affecting flesh pigmentation, and white peaches had high *CCD4* transcription abundance [16,17]. These studies provided unequivocal evidence for the critical role of *CCD4* in carotenoid accumulation. In the present study, *NpCCD4* exhibited remarkably different expression levels between SW and PZ. The *NpCCD4* gene had an extremely high expression level in SW white perianth and pale yellow corona, but was hardly expressed in PZ. We also provided results for earlier developmental stages during pigment formation (S1 to S3) as well (Figure 6C), and *CCD4* expressed highly in SW compared with PZ, which mean that in the whole flowering period the carotenoid synthesis was accompanied with carotenoid degradation. Our results were similar to those in chrysanthemum [15], with the transcriptional level of most carotenoid biosynthetic genes being similar in white and yellow petals, *CmCCD4a* was highly expressed in white petals as compared with that in yellow petals. Meanwhile, the expression profile of *NpCCD4* in F1 also showed the regulation of high expression in white-perianth hybrids and hardly expressed in yellow-perianth hybrids. By silencing the *NpCCD4* gene, the corona of SW became orange and no longer fading, demonstrating that CCD4 was indeed the cause of the degradation of carotenoids in SW. These findings indicate that *NpCCD4* is the important factor that causes the low carotenoid content and color fading phenomenon of SW by mediating carotenoid turnover.

### 3.4. Substrates and Products of NpCCD4

Due to the pivotal role of CCDs in the accumulation of carotenoids, their functions had been extensively studied. Unlike other CCDs, CCD4 have wide substrate specificities, from the upstream β-carotene to downstream neoxanthin [19]. CCD4 enzymes, which had cleavage activity at the C9–C10 and/or the C9’–C10’ double bond, could cleavage β-carotene and produce β-ionone in *Crocus* [18], *Chrysanthemum* [15], and apple [14], and cleavage all-*trans*-β-carotene, leading to the production of all-*trans*-β-apo-10′-carotenal and β-ionone in potato [40]. While, Citrus CCD4 enzymes cleave β-carotene, β,β-cryptoxanthin, and zeaxanthin at the C7′–C8′ double bond, leading to β-apo-8′-carotenal and β-citraurin, and two volatile compounds: cyclocitral and 3-OH-cyclocitral [41]. A recent report on *Arabidopsis thaliana* CCD4 analyzed the substrates and products of AtCCD4 enzymes in detail through in vitro assays and dynamic modeling. The results showed that AtCCD4 had the activity to convert all-*trans*-β-carotene into product all-*trans*-β-apo-10′-carotenal and β-Ionone, and cleave β,β-cryptoxanthin and zeaxanthin at the C9–C10 or the C9′–C10′ double bond yielding high amounts of 3-OH-β-apo-10′-carotenal, but it could not cleavage 9-*cis*-violaxanthin and all-*trans*-neoxanthin [28].

In our study, all the carotenoids decreased sharply after the S4 stage in SW, except for all-*trans*-neoxanthin and 9-*cis*-neoxanthin, indicating that NpCCD4 does not have cleavage activity on these two carotenoids either. The decreased carotenoids include β-carotene, cryptoxanthin, zeaxanthin, violaxanthin, antheraxanthin, and lutein, among which β-carotene is the precursor of cryptoxanthin, zeaxanthin, violaxanthin, and antheraxanthin. However, in the carotenoid synthesis pathway there is a metabolite-dependent feedback regulation for the content of carotenoids and neoxanthin is located downstream of the carotenoid biosynthesis pathway. Thus, we could not deduce the exact substrate of NpCCD4 by the change and composition of carotenoids in *N. pseudonarcissus*. In the VIGS assay, the TC increased significantly in the CCD-silenced coronas as compared with the control, with the most noticeable increase in all-*trans*-β-carotene. These data indicate that all-*trans*-β-carotene is the likely one of substrate of NpCCD4 in planta. At the same time, other carotenoids, such as cryptoxanthin, zeaxanthin, and violaxanthin, also have the possibility of being degraded by NpCCD4. Unfortunately, carotenoid enzymes are labile in vitro, making the study on enzyme activity difficult using the classic biochemical approaches. Further experiments need to be carried out to determine the specific substrates and products of NpCCD4. In many cases, the cleavage of carotenoid by CCDs produces various volatiles (e.g., β-ionone, β-citraurin, etc.). However, we did not detect any β-ionone or β-citraurin in the volatile compound of perianths and coronas of SW, which should be detected by HS-SPME/GC-MS if existing [42]. We can conclude that the cleavage product of CCD degradation of carotenoids does not conclude β-ionone or β-citraurin, but the exact substrates and products of NpCCD4 in planta need further verification.

## 4. Materials and Methods

### 4.1. Plant Materials

‘Slim Whitman’ has white perianth and light yellow corona matured to white; ‘Pinza’ has yellow perianth and yellow-orange corona (Figure 1). They are both large-cupped daffodils and possess the same basic chromosome numbers (2*n* = 28) [43]. The voucher specimen numbers and phenotypic characterization of ‘Slim Whitman’ and ‘Pinza’ were listed in Supplementary Table S5 (http://daffseek.org/). Cross-breeding between ‘Slim Whitman’ and ‘Pinza’ were performed and 27 reciprocal hybrids with different genotypes were bred [3]. SW, PZ, and the 27 reciprocal hybrids were planted in horticultural farm of Shanghai Jiao Tong University, Shanghai, China.

### 4.2. Evaluation of Carotenoids

Extraction and evaluation of carotenoids were performed as previously described [7]. Standard of β-carotene was purchased from Sigma-Aldrich China (Shanghai, China). The data of carotenoids contents were an average value of three biological replicates.

### 4.3. Sampling and RNA Extraction

Two biological replications of perianth of ‘Slim Whitman’ (SWP-1 and SWP-2), corona of ‘Slim Whitman’ (SWC-1 and SWC-2), perianth of ‘Pinza’ (PZP-1 and PZP-2), and corona of ‘Pinza’ (PZC-1 and PZC-2) were collected at the S4 flowering stage in March of 2017. All eight samples were immediately frozen in liquid nitrogen and stored at −80 °C for further analysis. High-quality total RNA from the samples was isolated using the RNAprep Pure Plant Kit (Tiangen, Beijing, China), following the manufacturer’s instructions. Subsequently, cDNA libraries were constructed from the total RNA of these eight samples.

### 4.4. RNA Sequencing and Transcriptome Assembly

The cDNA libraries were sequenced using an Illumina HiSeq 4000 platform, and paired-end reads were generated by Biomarker Co. (Beijing, China). High-quality reads were assembled into candidate unigenes by the Trinity program (Available online: http://trinityrnaseq.sourceforge.net/) [44], as described in the previous article [45].

### 4.5. Bioinformatic Analysis

Assembled unigenes were annotated using BLAST alignment against public databases, including NR (Available online: http://www.ncbi.nlm.nih.gov), Pfam (Available online: http://pfam.sanger.ac.uk/), Swiss-Prot (Available online: http://www.expasy.ch/sprot), String (Available online: http://string-db.org/), and the KEGG (Available online: http://www.genome.jp/kegg) with an E value cut off of 10^−5^ [44,46]. Blast2go (Available online: http://www.blast2go.com/b2ghome) was carried out to perform GO (Available online: Gene Ontology, http://www.geneontology.org) annotation [47]. COG (Available online: http://www.ncbi.nlm.nih.gov/COG/) was carried out to perform COG classifications.

The Pearson’s correlation coefficient was used as an evaluation index for correlation between samples [48]. The correlation evaluation between biological replications was contained in Supplementary Table S6. Sample correlation analysis showed that sampling was scientific and repeatable and the obtained DEGs are reliable.

Gene expression levels were estimated by RSEM [49] in the eight samples. The expression level of each unigene (FPKM) was calculated [49] and the differentially expressed genes (DEGs) were screened with the threshold false discovery rate (FDR) ≤0.001 and the absolute value of log2 Fold Change ≥4 by DESeq R package (1.10.1) (Available online: http://www.bioconductor.org/packages/release/bioc/html/DESeq.html).

Gene Ontology (GO) enrichment analysis of the DEGs was implemented by the topGO R packages based on the Kolmogorov–Smirnov test. KOBAS software (Available online: http://kobas.cbi.pku.edu.cn/help.do) [50] was utilized to analyze the statistical enrichment of DEGs in KEGG pathways.

### 4.6. Quantitative Real-Time PCR

The total RNA used for qRT-PCR analysis were extracted from perianths and coronas at S1 to S7 and scapes, leaves, bulbs at S4, using the RNAprep Pure Plant Kit (Tiangen, Beijing, China), following the manufacturer’s instructions. Afterwards, total RNA were converted into first strand cDNA using the SuperScriptTM II First-Strand Synthesis Kit (Takara Biomedical Technology Co., Beijing, China). Nine carotenoids-related genes were selected for verification of the sequencing results by quantitative real-time PCR (qRT-PCR) with primers that were designed by Primer Premier 5 software (Available online: www.premierbiosoft.com) (Supplementary Table S7). SYBR Green qPCR Master Mix was used for the detection of PCR products (Tiangen Biotech, Beijing, China) according to the manufacturer’s instructions with three technical replicates and three biological replicates. In our previous work, *Actin* was demonstrated to be one of the most stable reference genes in *N. pseudonarcissus* [51] and used as the internal control for normalization. The relative expression levels of the selected unigenes were calculated by the 2^−ΔΔ*C*t^ method [52].

### 4.7. Silencing of NtCCD4 in Coronas of Slim Whitman by VIGS

The silencing of *NtCCD4* in coronas of SW by VIGS was carried out according to the procedures that are described by Dai et al. with minor modifications [53]. For the construction of the pTRV2-CCD4 vector, a 425-bp NpCCD4 fragment was recombined into the pTRV2 vector by the ClonExpress II One Step Cloning Kit (C112-01, Vazyme, Nanjing, China). Recombinant primers were designed by CE Design software (Available online: http://www.vazyme.com). The bacterial suspension solution with *Agrobacterium tumefaciens* containing tobacco rattle virus (TRV)-NpCCD4 or TRV control was injected into the unopened coronas (S1, when the flower buds were standing straight and could hold the bacterial suspension solution). By the time of flowering (about seven days after injection), the chromaticity of coronas from five biological replications were measured by CIE 1976 L* a* b* (CIELAB) system, which describes flower color by L*, a*, and b* parameters [54]. Primers that are used for vector construction and detection are indicated in Supplementary Table S7.

## 5. Conclusions

In conclusion, by parallel metabolomic and transcriptomic analyses, we found that the NpCCD4 mediated carotenoid degradation is one of the crucial factors that causes the low carotenoid content and color fading phenomenon in the white-perianth *N. pseudonarcissus* cultivar. Our results strongly suggested that regulating the expression of *NpCCD4* was a potential breeding strategy to obtain the *N. pseudonarcissus* flower with different colors. Besides, the research provided mass RNA-seq data and new insights into carotenoid metabolism in *N. pseudonarcissus*.

## Figures and Tables

**Table 1:** Summary of functional annotation for *N. pseudonarcissus* unigenes in public databases.

Anno_Database	Annotated_Number	300 ≤ Length < 1000	Length ≥ 1000
COG_Annotation	8896	1908	5171
GO_Annotation	16,349	5409	8338
KEGG_Annotation	9992	3291	5268
KOG_Annotation	16,454	5254	8650
Pfam_Annotation	18,752	5172	11,144
Swissprot_Annotation	17,188	5632	9445
eggNOG_Annotation	27,475	9207	13,423
nr_Annotation	28,304	9983	13,754
All_Annotated	30,156	10,392	13,845

**Table 2:** The number of differentially expressed genes between different databases.

DEG_Set	All_DEG	Up-Regulated	Down-Regulated
SWP vs. SWC	730	352	378
SWP vs. PZP	1022	511	511
SWC vs. PZC	3880	2232	1648
PZP vs. PZC	3317	1636	1681

SWP: perianth of ‘Slim Whitman’; SWC: corona of ‘Slim Whitman’; PZP: perianth of ‘Pinza’; PZC: corona of ‘Pinza’.

## Supplementary Materials

The following are available online at http://www.mdpi.com/1422-0067/19/12/4006/s1, Table S1: The mean content (μg/g) of carotenoid compounds in perianths and coronas of SW and PZ in different stages; Table S2: The mean content (μg/g) of carotenoid compounds in perianths and coronas of 27 hybrids of ‘Slim Whitman’ and ‘Pinza’; Table S3: Assessment of Illumina transcriptome sequencing analysis; Table S4: The number of differentially expressed genes annotated in different databases; Table S5: Sources and phenotypic characterization of ‘Slim Whitman’ and ‘Pinza’. Table S6: Correlation evaluation between samples; Table S7: Primers used in qRT-PCR, vector construction and RT-PCR analysis; Figure S1: The HPLC chromatogram of carotenoids from the perianths and coronas; Figure S2: Volcano plot of the differentially expressed genes (DEGs) between SWP and SWC (A), SWP and PZP (B), SWC and PZC (C), PZP and PZC (D); Figure S3: DEGs annotated with GO classification. (A): SWP and SWC; (B): SWP and PZP; (C): SWC and PZC; (D): PZP and PZC; Figure S4: DEGs annotated with COG classification. (A): SWP and SWC; (B): SWP and PZP; (C): SWC and PZC; (D): PZP and PZC; Figure S5: The enrichment analysis based on KEGG pathways of DEGs. (A): SWP and SWC; (B): SWP and PZP; (C): SWC and PZC; (D): PZP and PZC; Figure S6: The relative expression of unigene c77684.graph_c0 (CCD) in different flowering stages and different tissue of SW and PZ; Figure S7: Clustalw tree analysis of c77684.graph_c0 (CCD) with the nine carotenoid dioxygenases of *Arabidopsis*; Figure S8: Alignment of the amino acid residues of NpCCD4 with CCD4 proteins from other plants.
