# Supplementary material for: Transcriptome Sequencing and Biochemical Analysis of Perianths and Coronas Reveal Flower Color Formation in Narcissus pseudonarcissus
Source: Int J Mol Sci. 2018 Dec 12;19(12):4006. doi: 10.3390/ijms19124006 (PMC6320829; doi:10.3390/ijms19124006)
Supplement: Supplementary file 1 [file ijms-19-04006-s001.zip › Supplementary Table S1,.docx]

**Table S1.** The mean content (μg/g) of carotenoid compounds in perianths and coronas of SW and PZ in different stages.

|  | all-trans-neoxanthin*^a^* | 9-cis-neoxanthin | all-trans-antheraxanthin | all-trans-lutein | 9-cis-violaxanthin | all-trans-zeaxanthin | all-trans-α-cryptoxanthin | all-trans-β-cryptoxanthin | all-trans-β-carotene | 9-cis-carotene | total |
| --- | --- | --- | --- | --- | --- | --- | --- | --- | --- | --- | --- |
| SWP 2*^b^* | ——*^c^* | 0.183 | 0.178 | 2.739 | 2.191 | 0.705 | 0.963 | 0.116 | 3.436 | 0.100 | 10.610 |
| SWC 2 | 2.008 | 4.232 | 2.513 | 9.610 | 7.668 | 0.659 | 2.988 | 1.444 | 3.519 | 0.448 | 35.089 |
| SWP 3 | —— | 0.166 | 0.305 | 3.768 | 3.253 | 0.322 | 1.975 | 0.017 | 3.618 | 0.149 | 13.573 |
| SWC 3 | 3.386 | 6.822 | 7.962 | 25.228 | 21.593 | 1.596 | 7.502 | 2.025 | 4.166 | 0.896 | 81.176 |
| SWP4 | 0.231 | 0.595 | 0.561 | 3.101 | 0.472 | —— | 0.598 | —— | 0.823 | —— | 6.381 |
| SWC 4 | 5.408 | 5.223 | 12.948 | 20.872 | 8.094 | 1.982 | 7.152 | 3.096 | 4.680 | 1.192 | 70.648 |
| SWP 5 | 0.207 | 0.148 | —— | 0.438 | —— | —— | 0.037 | —— | —— | —— | 0.830 |
| SWC 5 | 7.999 | 7.592 | 1.223 | —— | 0.052 | —— | 0.072 | 0.188 | —— | —— | 17.128 |
| SWP 6 | —— | —— | —— | —— | —— | —— | —— | —— | —— | —— | 0.090 |
| SWC 6 | 1.680 | 1.698 | 0.225 | 0.118 | —— | —— | —— | 1.101 | —— | —— | 4.822 |
| SWP 7 | —— | —— | —— | —— | —— | —— | —— | —— | —— | —— | 0.327 |
| SWC 7 | —— | —— | —— | 0.816 | —— | —— | 0.132 | 0.804 | —— | —— | 1.751 |
| PZP 2 | 0.548 | 0.465 | 0.885 | 7.303 | 6.340 | 0.985 | 2.224 | 0.183 | 4.664 | 0.282 | 23.878 |
| PZC 2 | 3.801 | 7.021 | 11.012 | 50.506 | 42.008 | 6.293 | 6.506 | 0.979 | 8.282 | 0.963 | 137.371 |
| PZP 3 | 2.174 | 0.315 | —— | 18.788 | 14.490 | 0.297 | —— | —— | 3.934 | 0.830 | 40.828 |
| PZC 3 | —— | —— | 15.254 | 33.527 | 25.992 | 8.752 | —— | 0.050 | 32.880 | 2.672 | 119.127 |
| PZP 4 | 2.506 | 5.378 | 3.568 | 32.515 | 27.867 | 0.595 | 0.880 | 0.332 | 6.307 | 1.012 | 80.959 |
| PZC 4 | 9.378 | 16.830 | 10.056 | 65.162 | 61.295 | 3.849 | 1.427 | 0.564 | 83.369 | 4.382 | 256.312 |
| PZP 5 | 31.411 | 33.545 | 2.315 | 101.208 | 2.118 | 0.058 | —— | 0.481 | 4.291 | 0.426 | 175.852 |
| PZC 5 | 70.774 | 65.642 | 13.564 | 221.818 | 2.108 | 2.504 | 1.660 | 14.804 | 59.239 | 7.854 | 459.968 |
| PZP 6 | 7.506 | 8.799 | 12.346 | 65.158 | 4.896 | 0.529 | 1.225 | 0.327 | 4.022 | 0.406 | 105.213 |
| PZC 6 | 23.468 | 23.380 | 22.852 | 122.482 | 5.664 | 2.272 | 2.711 | 4.216 | 64.956 | 4.850 | 276.852 |
| PZP 7 | 16.439 | 18.314 | 8.342 | 88.299 | 2.192 | 0.169 | 0.087 | 0.109 | 2.472 | 0.006 | 136.429 |
| PZC 7 | 38.606 | 39.296 | 3.333 | 91.541 | 0.507 | 0.842 | 0.592 | 5.188 | 141.407 | 2.888 | 324.200 |

*^a^*Carotenoid compounds detected in narcissus flowers; *^b^* SWP: perianth of SW. SWC: corona of SW. PZP: perianth of PZ. PZC: corona of PZ. Number 2 to 7 represents stage2 to stage 7. *^c^* ug/g: ug carotenoid compounds per gram fresh weight. —: Carotenoid compounds didn’t exist or under the detection line.
