# Supplementary material for: Transcriptome Sequencing and Biochemical Analysis of Perianths and Coronas Reveal Flower Color Formation in Narcissus pseudonarcissus
Source: Int J Mol Sci. 2018 Dec 12;19(12):4006. doi: 10.3390/ijms19124006 (PMC6320829; doi:10.3390/ijms19124006)
Supplement: Supplementary file 1 [file ijms-19-04006-s001.zip › Supplementary Table S2,.docx]

**Table S2.** The mean content (μg/g) of carotenoid compounds in perianths and coronas of 27 hybrids of ‘Slim Whitman’ and ‘Pinza’.

|  | all-trans-neoxanthin | 9-cis-neoxanthin | all-trans-antheraxanthin | all-trans-lutein | 9-cis-violaxanthin | all-trans-zeaxanthin | all-trans-α-cryptoxanthin | all-trans-β-cryptoxanthin | all-trans-β-carotene | 9-cis-carotene | TC*^a^* |
| --- | --- | --- | --- | --- | --- | --- | --- | --- | --- | --- | --- |
| **Perianths** | |  |  |  |  |  |  |  |  |  |  |
| **Yellow perianths** | |  |  |  |  |  |  |  |  |  |  |
| SP11-P | 6.42*^b^* | 3.42 | 0.67 | 14.44 | 9.31 | ——*^c^* | —— | —— | 0.76 | —— | 35.02 |
| SP12-P | 3.20 | 5.84 | 0.66 | 38.09 | 32.55 | —— | —— | —— | 2.14 | 0.40 | 82.88 |
| SP13-P | 1.24 | 0.68 | 0.22 | 2.76 | 1.46 | —— | 1.78 | 4.95 | 3.88 | —— | 16.96 |
| SP14-P | 0.86 | 1.13 | 0.63 | 18.11 | —— | —— | —— | 0.37 | 0.46 | —— | 21.55 |
| SP15-P | 0.17 | 1.08 | —— | 8.51 | 2.17 | —— | —— | 0.13 | 8.37 | —— | 20.43 |
| PS08-P | 2.36 | 0.38 | —— | 34.89 | 24.41 | —— | —— | 2.26 | 2.29 | 0.13 | 66.72 |
| PS09-P | 6.62 | 6.17 | 2.15 | 24.15 | 20.08 | 0.40 | —— | 0.13 | 1.08 | 0.22 | 61.01 |
| PS10-P | 18.52 | 13.13 | 1.88 | 1.54 | 0.70 | 0.41 | —— | —— | 2.17 | 0.38 | 38.74 |
| PS11-P | 0.10 | 0.51 | 3.89 | 9.73 | 5.11 | 5.09 | 0.53 | 0.10 | 0.56 | —— | 25.62 |
| PS12-P | 6.29 | 3.85 | 1.24 | 4.12 | 2.42 | 0.34 | —— | 0.02 | 2.85 | 0.41 | 21.54 |
| **White perianths** | |  |  |  |  |  |  |  |  |  |  |
| SP01-P | 0.68 | 0.96 | 0.38 | 5.56 | 2.29 | —— | —— | —— | —— | —— | 7.85 |
| SP02-P | 0.13 | 0.55 | —— | —— | 2.12 | —— | 0.13 | 0.70 | 0.05 | —— | 3.00 |
| SP03-P | —— | —— | 3.57 | 1.36 | 1.08 | 0.59 | 0.05 | —— | —— | —— | 3.08 |
| SP04-P | —— | —— | —— | 0.03 | —— | —— | 0.55 | 0.93 | —— | —— | 1.51 |
| SP05-P | —— | —— | —— | —— | —— | —— | 0.37 | 0.66 | —— | —— | 1.03 |
| SP06-P | —— | —— | —— | 0.41 | 0.28 | —— | 0.02 | —— | 0.08 | —— | 0.80 |
| SP07-P | —— | 0.02 | —— | 0.53 | 0.40 | —— | 0.83 | 0.55 | 0.38 | —— | 2.69 |
| SP08-P | 0.24 | 0.04 | —— | 0.98 | 0.34 | —— | —— | 0.54 | 0.66 | —— | 2.52 |
| SP09-P | 1.08 | 0.30 | —— | 1.05 | 0.45 | —— | 3.47 | 2.27 | 0.37 | —— | 7.60 |
| SP10-P | 0.00 | —— | —— | 0.10 | —— | —— | 0.30 | 0.25 | —— | —— | 0.65 |
| PS01-P | —— | —— | —— | 0.88 | —— | —— | 1.31 | —— | 2.04 | —— | 4.23 |
| PS02-P | 1.10 | 0.37 | 0.72 | 0.23 | —— | —— | —— | —— | 1.53 | 0.03 | 1.79 |
| PS03-P | —— | —— | —— | 0.43 | —— | —— | 0.76 | 0.13 | 0.18 | —— | 1.51 |
| PS04-P | —— | —— | —— | —— | —— | —— | 1.29 | 1.06 | —— | —— | 2.36 |
| PS05-P | —— | —— | —— | —— | 0.61 | —— | 0.30 | 0.05 | 3.04 | 0.05 | 4.05 |
| PS06-P | _ | —— | —— | —— | 0.20 | —— | 1.74 | 0.95 | 0.15 | —— | 3.04 |
| PS07-P | —— | —— | —— | 0.45 | —— | —— | 0.07 | 0.30 | 0.25 | —— | 1.06 |
| **Coronas** | |  |  |  |  |  |  |  |  |  |  |
| **Yellow coronas** | |  |  |  |  |  |  |  |  |  |  |
| PS01-C | 0.70 | 0.41 | —— | 6.54 | 2.38 | —— | 0.56 | 0.37 | 0.83 | —— | 11.79 |
| PS02-C | 32.22 | 21.39 | 1.91 | 64.17 | 48.18 | —— | 1.81 | 0.65 | 1.01 | —— | 171.33 |
| PS04-C | 3.50 | 3.27 | 0.44 | 3.15 | 0.68 | —— | 0.20 | - | 2.26 | 0.40 | 13.90 |
| PS05-C | 1.38 | 2.34 | 0.92 | 3.62 | 1.58 | 0.11 | —— | —— | 1.56 | 0.35 | 11.86 |
| PS06-C | 2.31 | 4.13 | 0.56 | 3.75 | 1.18 | 0.50 | —— | —— | 1.68 | 0.30 | 14.40 |
| PS07-C | —— | 1.38 | 0.16 | 4.92 | 2.28 | 0.87 | —— | —— | 1.44 | 0.25 | 11.31 |
| PS09-C | 4.91 | 4.86 | 1.32 | 0.10 | 24.43 | 0.54 | —— | —— | 5.86 | 0.85 | 42.88 |
| PS11-C | 3.75 | 3.42 | 10.67 | 12.40 | 8.68 | —— | 0.12 | —— | 3.73 | 0.50 | 43.27 |
| PS12-C | 0.91 | 1.84 | —— | 6.61 | 2.17 | —— | —— | —— | 0.41 | —— | 11.95 |
| SP01-C | 2.20 | 1.02 | —— | 5.28 | 0.95 | —— | 1.28 | 1.01 | —— | —— | 11.74 |
| SP02-C | 0.95 | —— | 0.22 | 6.57 | 1.10 | —— | 1.84 | —— | 0.24 | 0.51 | 11.44 |
| SP03-C | 0.80 | 1.98 | 0.12 | 2.15 | 0.97 | —— | 0.93 | 1.36 | 3.89 | 0.30 | 12.49 |
| SP05-C | 1.53 | 2.49 | —— | 0.17 | —— | 0.02 | 0.61 | —— | 8.58 | 0.51 | 13.91 |
| SP06-C | 0.68 | 1.28 | 2.37 | 3.72 | 3.57 | 0.67 | 3.29 | 1.41 | 1.43 | 0.20 | 18.61 |
| SP07-C | 1.33 | 2.39 | 0.23 | 5.34 | 1.83 | 0.83 | —— | —— | 2.39 | —— | 14.34 |
| SP08-C | 0.78 | 0.86 | 0.22 | 8.61 | 1.07 | 0.22 | —— | —— | —— | —— | 11.76 |
| SP09-C | 8.78 | 6.12 | 0.65 | —— | 1.11 | —— | 2.26 | 1.24 | 13.03 | 1.01 | 34.21 |
| SP10-C | 0.73 | 2.12 | 0.34 | 4.68 | 2.48 | 0.23 | —— | —— | 1.94 | 0.37 | 12.89 |
| SP11-C | 33.41 | 18.36 | 3.84 | 17.79 | 13.84 | —— | 3.12 | 0.66 | 4.45 | 0.91 | 96.39 |
| SP12-C | 33.29 | 22.02 | 0.08 | 77.81 | 56.18 | 0.77 | 0.71 | —— | 5.05 | 1.33 | 197.25 |
| SP13-C | 17.07 | 11.40 | 1.81 | 26.65 | 17.95 | 0.97 | —— | —— | 10.83 | 2.97 | 89.64 |
| SP14-C | 3.35 | 3.54 | 1.35 | —— | 23.04 | —— | 0.48 | 0.50 | 1.39 | 0.17 | 33.81 |
| SP15-C | 3.35 | 2.06 | 0.53 | 6.80 | 3.83 | —— | 0.08 | —— | 3.10 | —— | 19.77 |
| **Coronas with orange rim** | | |  |  |  |  |  |  |  |  |  |
| PS03-C | 0.28 | 0.80 | —— | 0.98 | —— | —— | —— | —— | 13.54 | 0.25 | 15.85 |
| PS08-C | 3.72 | 1.03 | 5.41 | 58.76 | 56.17 | 2.19 | —— | —— | 29.50 | 1.77 | 158.54 |
| PS10-C | 5.03 | 5.05 | 1.37 | 6.24 | 22.34 | 0.14 | —— | —— | 14.92 | 0.40 | 55.49 |
| SP04-C | 1.79 | 3.42 | 0.46 | 0.71 | —— | 1.45 | 0.20 | —— | 35.37 | 0.51 | 43.92 |

*a:* TC, total content; *b:* μg/g: μg carotenoid compounds per gram fresh weight. *c:* —, Carotenoid compounds didn’t exist or under the detection line.
