# Supplementary material for: Transcriptome Sequencing and Biochemical Analysis of Perianths and Coronas Reveal Flower Color Formation in Narcissus pseudonarcissus
Source: Int J Mol Sci. 2018 Dec 12;19(12):4006. doi: 10.3390/ijms19124006 (PMC6320829; doi:10.3390/ijms19124006)
Supplement: Supplementary file 1 [file ijms-19-04006-s001.zip › Supplementary Table S3,.docx]

**Table S3.** Assessment of Illumina transcriptome sequencing analysis.

| Samples | Read Number | Base Number | GC Content(%) | Q30(%) | Accession number |
| --- | --- | --- | --- | --- | --- |
| SWP-1 | 26,176,498 | 7,698,124,016 | 49.46 | 89.48 | SRR8115250 |
| SWP-2 | 26,511,140 | 7,818,699,784 | 49.19 | 89.63 | SRR8115251 |
| SWC-1 | 35,050,166 | 10,297,954,424 | 49.52 | 89.98 | SRR8115248 |
| SWC-2 | 23,898,854 | 7,016,035,702 | 49.66 | 89.81 | SRR8115249 |
| PZP-1 | 34,436,684 | 10,115,031,292 | 49.39 | 89.56 | SRR8115254 |
| PZP-2 | 25,513,814 | 7,522,796,398 | 48.87 | 89.48 | SRR8115255 |
| PZC-1 | 30,109,875 | 9,011,080,018 | 45.93 | 93.01 | SRR8115252 |
| PZC-2 | 25,562,989 | 7,649,586,346 | 45.94 | 94.00 | SRR8115253 |

Read Number： pair-end Reads number in Clean Data; Base Number：total base number in Clean Data; GC Content：the percentages of G and C in Clean Data constitute the total base; Q30：the percentage of bases that Clean Data quality is greater than or equal to 30. Accession number: accession number of all raw data in the Short Read Archive (SRA) Sequence Data base in the National Center for Biotechnology Information (NCBI).
