# Supplementary material for: Transcriptome Sequencing and Biochemical Analysis of Perianths and Coronas Reveal Flower Color Formation in Narcissus pseudonarcissus
Source: Int J Mol Sci. 2018 Dec 12;19(12):4006. doi: 10.3390/ijms19124006 (PMC6320829; doi:10.3390/ijms19124006)
Supplement: Supplementary file 1 [file ijms-19-04006-s001.zip › Supplementary Table S4,.docx]

**Table S4.** The number of differentially expressed genes annotated in different databases.

| **DEG_Set** | **Annotated** | **COG** | **GO** | **KEGG** | **KOG** | **Pfam** | **Swiss-Prot** | **eggNOG** | **nr** |
| --- | --- | --- | --- | --- | --- | --- | --- | --- | --- |
| SWP vs SWC | 618 | 188 | 359 | 171 | 298 | 510 | 466 | 587 | 610 |
| SWP vs PZP | 632 | 161 | 321 | 170 | 315 | 454 | 416 | 564 | 613 |
| SWC vs PZC | 2202 | 556 | 1282 | 690 | 1112 | 1513 | 1434 | 2041 | 2156 |
| PZP vs PZC | 2305 | 685 | 1440 | 758 | 1155 | 1685 | 1570 | 2175 | 2274 |

DEG Set: names of the compared libraries; Annotated: the number of differentially expressed genes annotated; Third to the last column indicating the number of differentially expressed genes annotated by the functional databases.
