# Supplementary material for: Transcriptome Sequencing and Biochemical Analysis of Perianths and Coronas Reveal Flower Color Formation in Narcissus pseudonarcissus
Source: Int J Mol Sci. 2018 Dec 12;19(12):4006. doi: 10.3390/ijms19124006 (PMC6320829; doi:10.3390/ijms19124006)
Supplement: Supplementary file 1 [file ijms-19-04006-s001.zip › Supplementary Table S5,.docx]

Table S5. Sources and phenotypic characterization of ‘Slim Whitman’ and ‘Pinza’.

|  | Voucher specimen number | Classification | Season | Height | Hybridizer | Year of register |
| --- | --- | --- | --- | --- | --- | --- |
| Slim Whitman | S20040SW | Large-Cupped | Early to Mid-Season | Standard  32.5 to 67.5 cm | D.P. de Graaf,  the Netherlands | 1988 |
| Pinza | S20010PI | Large-Cupped | Mid-Season | Tall  greater than 67.5 cm | J. Lionel Richardson,  Ireland | 1962 |
