# Supplementary material for: Transcriptome Sequencing and Biochemical Analysis of Perianths and Coronas Reveal Flower Color Formation in Narcissus pseudonarcissus
Source: Int J Mol Sci. 2018 Dec 12;19(12):4006. doi: 10.3390/ijms19124006 (PMC6320829; doi:10.3390/ijms19124006)
Supplement: Supplementary file 1 [file ijms-19-04006-s001.zip › Supplementary Table S6,.docx]

**Table S6.** Correlation evaluation between samples.

| Sample | SWP-1 | SWP-2 | SWC-1 | SWC-2 | PZP-1 | PZP-2 | PZC-1 | PZC-2 |  |
| --- | --- | --- | --- | --- | --- | --- | --- | --- | --- |
| SWP-1 | 1*^a^* | 0.9672 | 0.6709 | 0.6775 | 0.8357 | 0.8393 | 0.2376 | 0.1859 |  |
| SWP-2 | 0.9672 | 1 | 0.7555 | 0.7611 | 0.7897 | 0.8131 | 0.2607 | 0.2025 |  |
| SWC-1 | 0.6709 | 0.7555 | 1 | 0.9928 | 0.517 | 0.5276 | 0.3522 | 0.2786 |  |
| SWC-2 | 0.6775 | 0.7611 | 0.9928 | 1 | 0.5228 | 0.5338 | 0.3651 | 0.2889 |  |
| PZP-2 | 0.8357 | 0.7897 | 0.517 | 0.5228 | 1 | 0.9529 | 0.2127 | 0.1618 |  |
| PZP-2 | 0.8393 | 0.8131 | 0.5276 | 0.5338 | 0.9529 | 1 | 0.2224 | 0.1685 |  |
| PZC-1 | 0.2376 | 0.2607 | 0.3522 | 0.3651 | 0.2127 | 0.2224 | 1 | 0.8963 |  |
| PZC-2 | 0.1859 | 0.2025 | 0.2786 | 0.2889 | 0.1618 | 0.1685 | 0.8963 | 1 |  |

***a,*** r^2: the closer the r^2 is to 1, the stronger the correlation between the two samples.
