# Supplementary material for: Transcriptome Sequencing and Biochemical Analysis of Perianths and Coronas Reveal Flower Color Formation in Narcissus pseudonarcissus
Source: Int J Mol Sci. 2018 Dec 12;19(12):4006. doi: 10.3390/ijms19124006 (PMC6320829; doi:10.3390/ijms19124006)
Supplement: Supplementary file 1 [file ijms-19-04006-s001.zip › Supplementary Table S7,.docx]

**Table S7.** Primers used in qRT-PCR, vector construction and RT-PCR analysis.

| Gene detected | Primer name | Sequence(5'to3') |
| --- | --- | --- |
| **For qRT-PCR** | | |
| GGPPS  (c94585.graph_c1) | NarGGPPS-L | AATTCGCCAAATGAAGCAAC |
|  | NarGGPPS-R | GCGACAAGACGACATACCA |
| PSY  (c100891.graph_c0) | NarPSY-L | TCAAGAGGGCAAGGATGT |
|  | NarPSY-R | GGCGGTCTCAATGATAAGG |
| PDS  (c100351.graph_c0) | NarPDS-L | GTTCTTCCCGCACCCTTAA |
|  | NarPDS-R | ACCCTGCCTTCTCATCCA |
| Z-ISO  (c92185.graph_c1) | NarZISO-L | GCGTCGATCGGTTTGATTGG |
|  | NarZISO-R | TTCTGGCGGCCATCAAGAAT |
| CRTISO  c98702.graph_c1 | NarCRTISO-L | ATCCTCGGTGATGTTTGG |
|  | NarCRTISO-R | TCGCTGTAGTCCCTATGT |
| LCYe  c97172.graph_c0 | NarLCYe-L | GCGTAGAATCGTTGGGAAA |
|  | NarLCYe-R | TGATTCTGCAGCGAGAGAGA |
| VED  c92832.graph_c0 | NarVED-L | CCGAGTGAAGAAACCACCAT |
|  | NarVED-R | CCGACCCATCAACTCTTGTT |
| CCD4  c77684.graph_c0 | NarCCD4-L | GGCAATGTCGTCGTCCTAAT |
|  | NarCCD4-R | TGTAGGAGGAAAGCGCAGTT |
| CYP97A  c94171.graph_c0 | Nar CYP97A -L | TCATGTGGTTGTCTCGCATT |
|  | Nar CYP97A -R | GGACACGACACACAATCTGG |
| ACT | NarACTINL2 | CGTGGTGGATCCTCAATTCT |
|  | NarACTINR2 | GGCATTGCAGATATGGCTTT |
| **For RT-PCR analysis** | | |
| CCD4 | NarCCD4-L | GGCAATGTCGTCGTCCTAAT |
|  | NarCCD4-R | TGTAGGAGGAAAGCGCAGTT |
| TRV2 | TRV2-L | GTATGTCAGTGATCGCAGTAG |
|  | TRV2-R | CGTCCGTTTAGACGCTTGCGTAGG |
| **For vector construction** | | |
| CCD4czTRV | CCD4czTRV-L | gtgagtaaggttaccgaattcTCTCCATCCATAATTAGCAGACAAA |
|  | CCD4czTRV-R | cgtgagctcggtaccggatccGAGTATCTGTAGGTATGGACGAAGCG |
