# Supplementary figures and images for: Transcriptome Sequencing and Biochemical Analysis of Perianths and Coronas Reveal Flower Color Formation in Narcissus pseudonarcissus
Source: Int J Mol Sci. 2018 Dec 12;19(12):4006. doi: 10.3390/ijms19124006 (PMC6320829; doi:10.3390/ijms19124006)

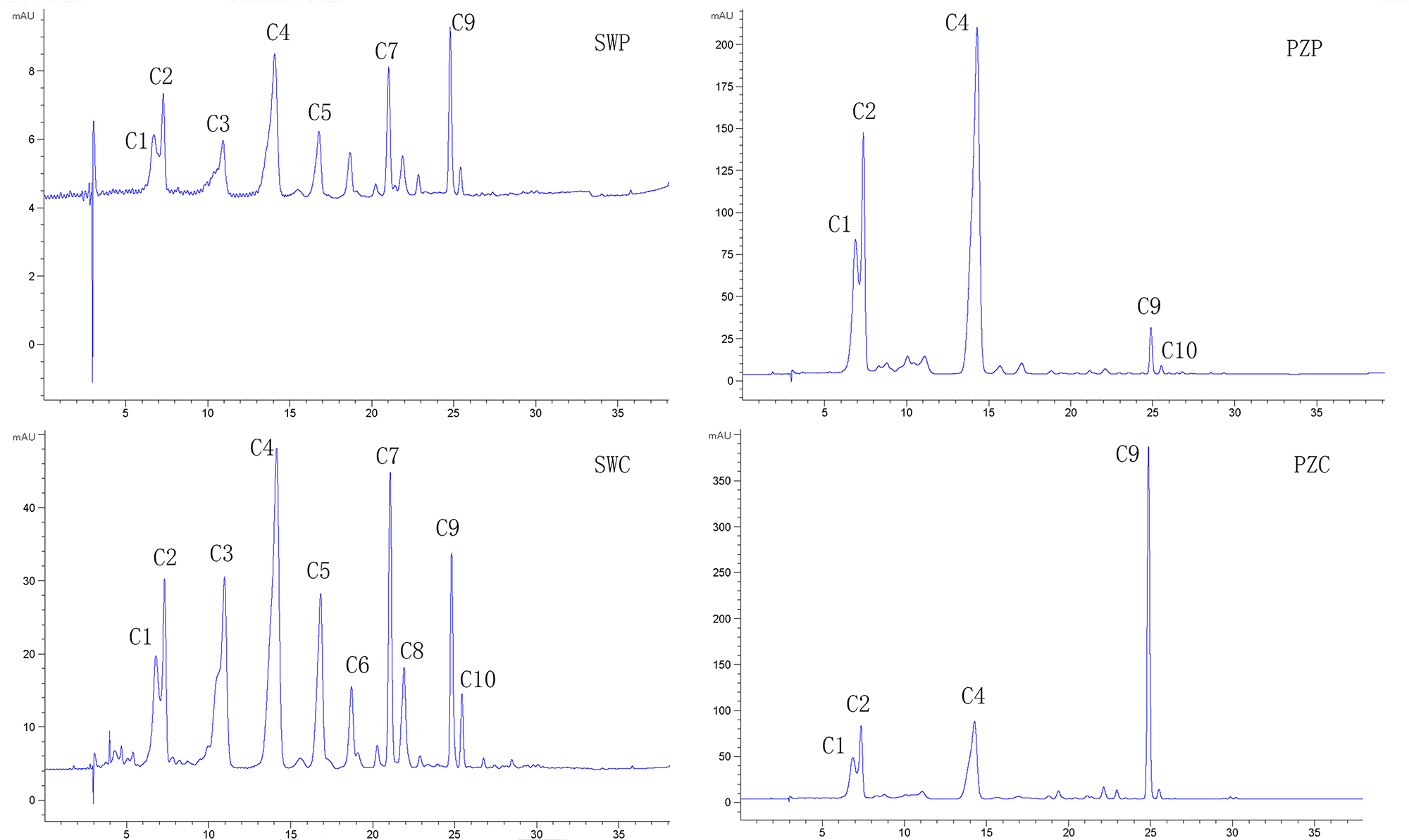

Supplement: Supplementary file 1 [file ijms-19-04006-s001.zip › Supplementary Fig. S1.tif]

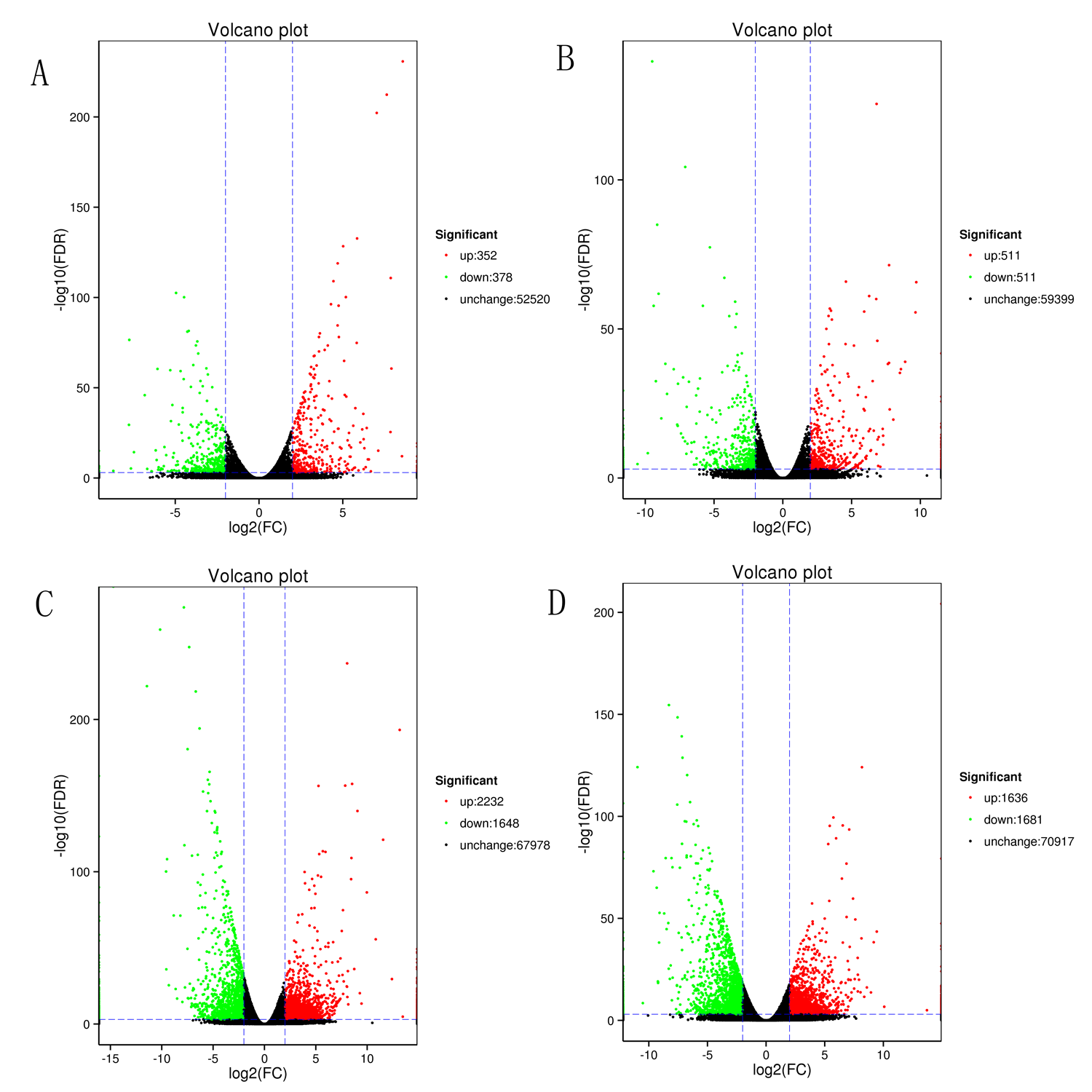

Supplement: Supplementary file 1 [file ijms-19-04006-s001.zip › Supplementary Fig. S2.tif]

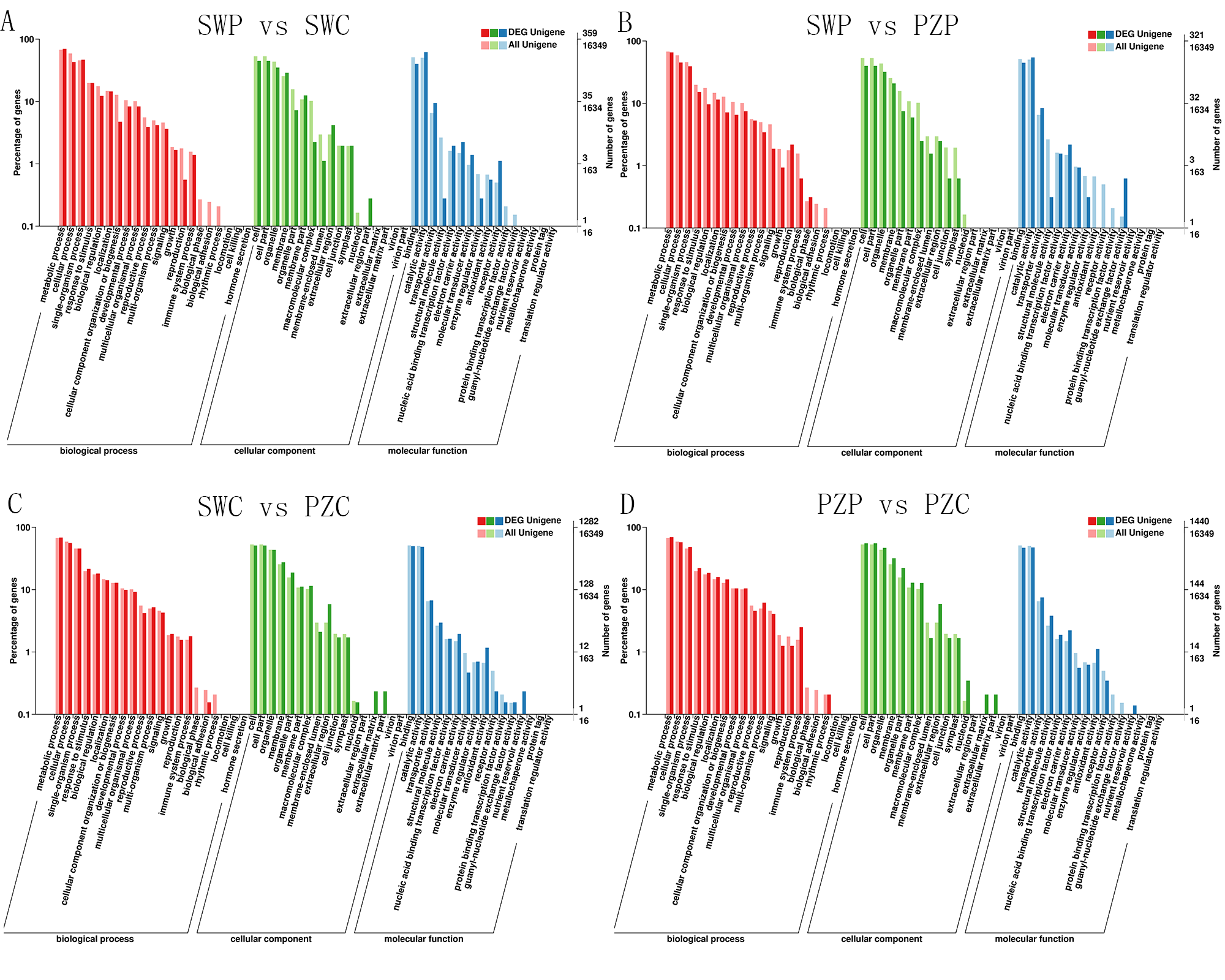

Supplement: Supplementary file 1 [file ijms-19-04006-s001.zip › Supplementary Fig. S3 .GO.tif]

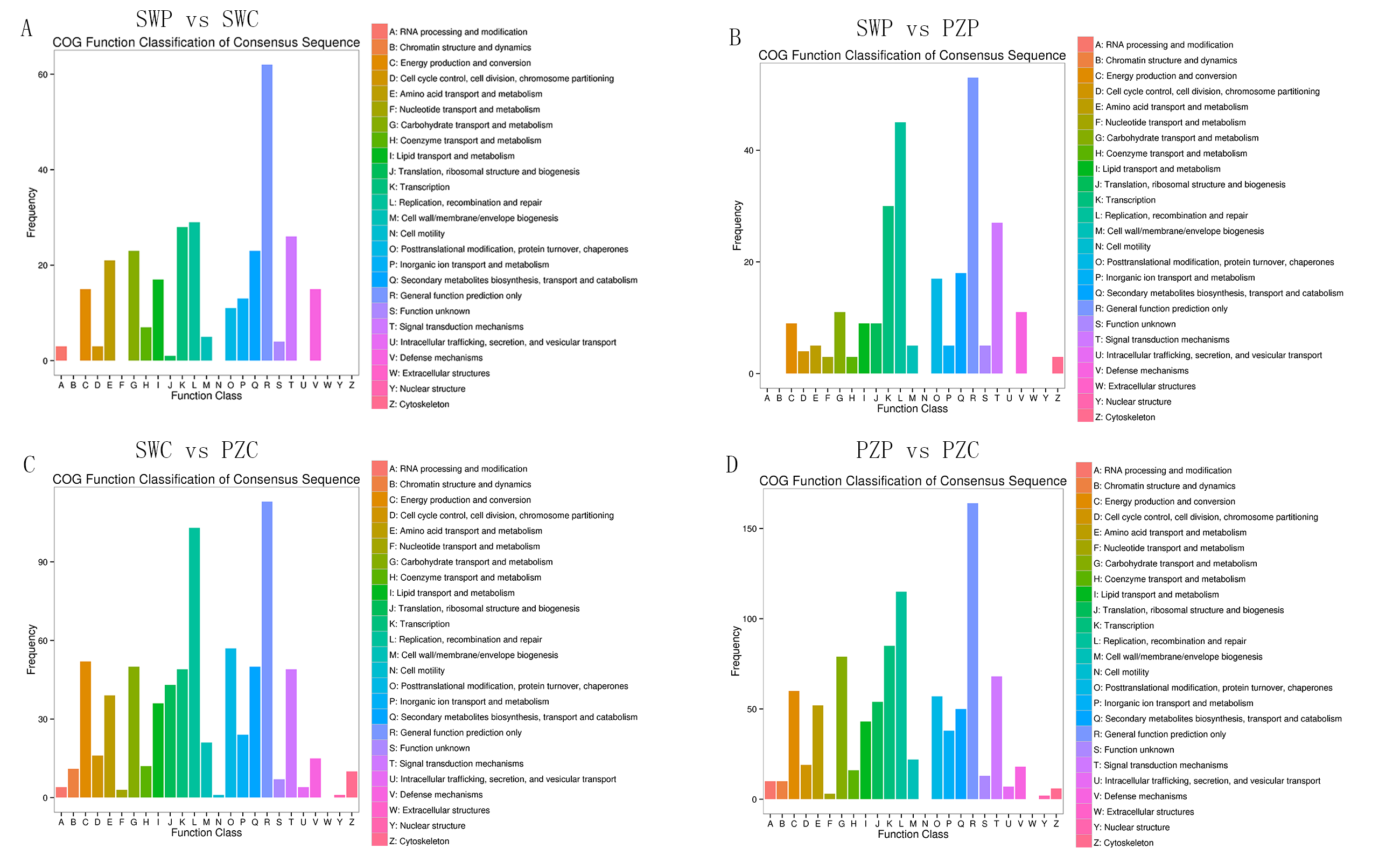

Supplement: Supplementary file 1 [file ijms-19-04006-s001.zip › Supplementary Fig. S4.COG.classification.tif]

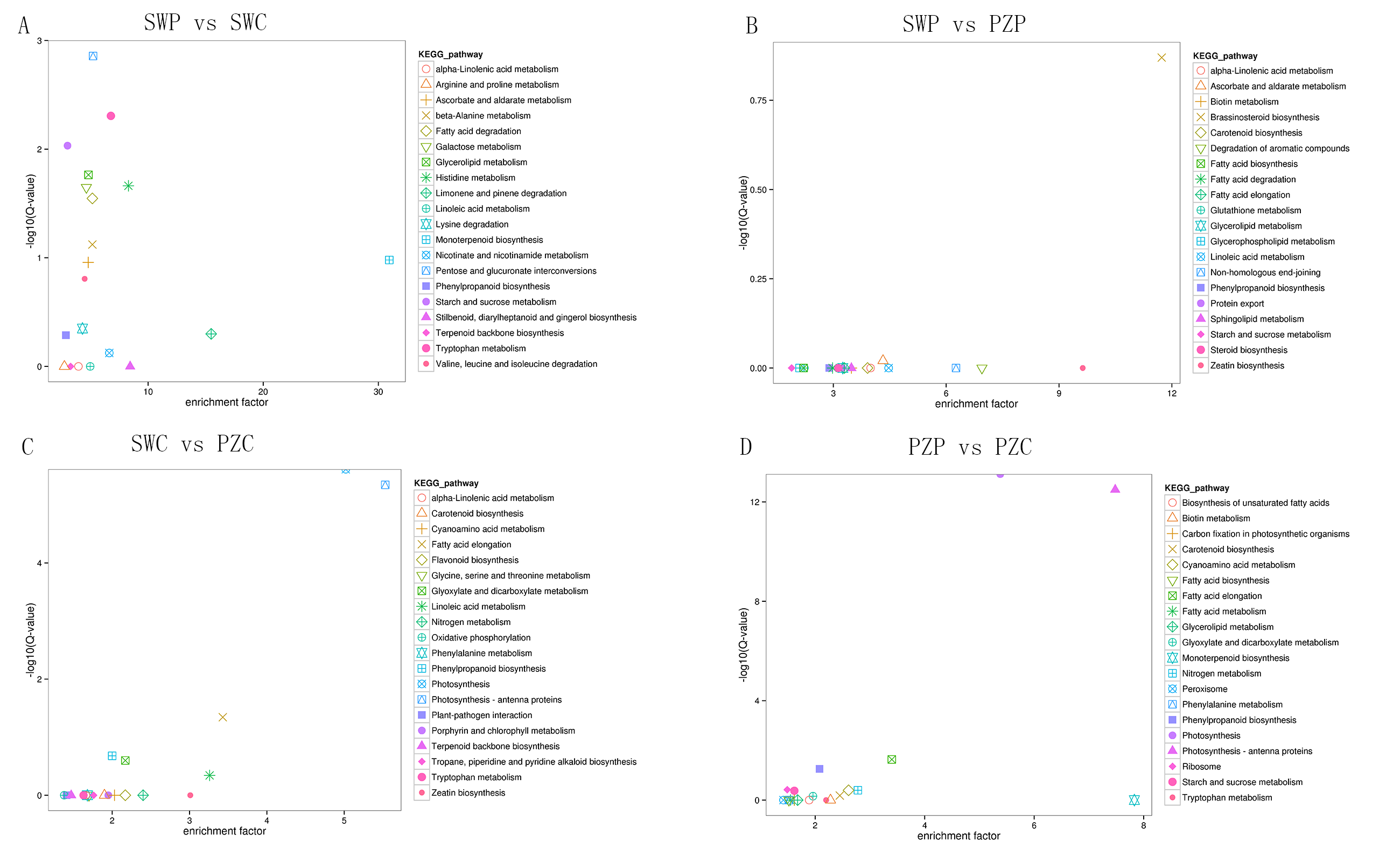

Supplement: Supplementary file 1 [file ijms-19-04006-s001.zip › Supplementary Fig. S5.tif]

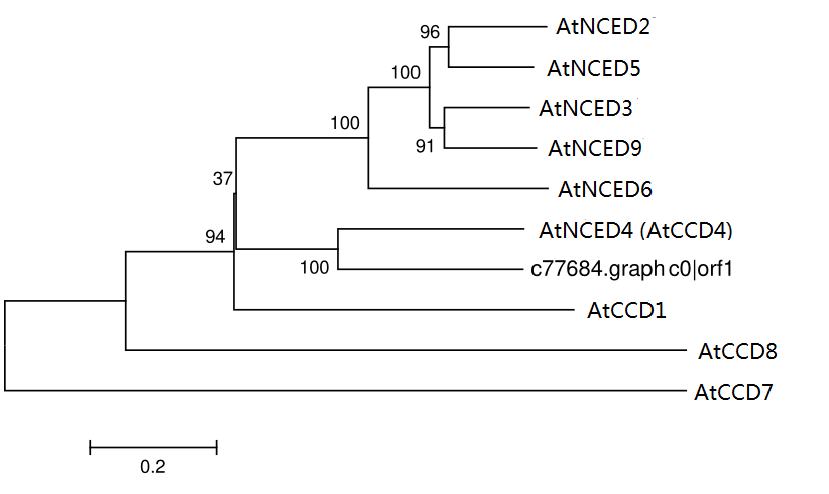

Supplement: Supplementary file 1 [file ijms-19-04006-s001.zip › Supplementary Fig. S7.tif]

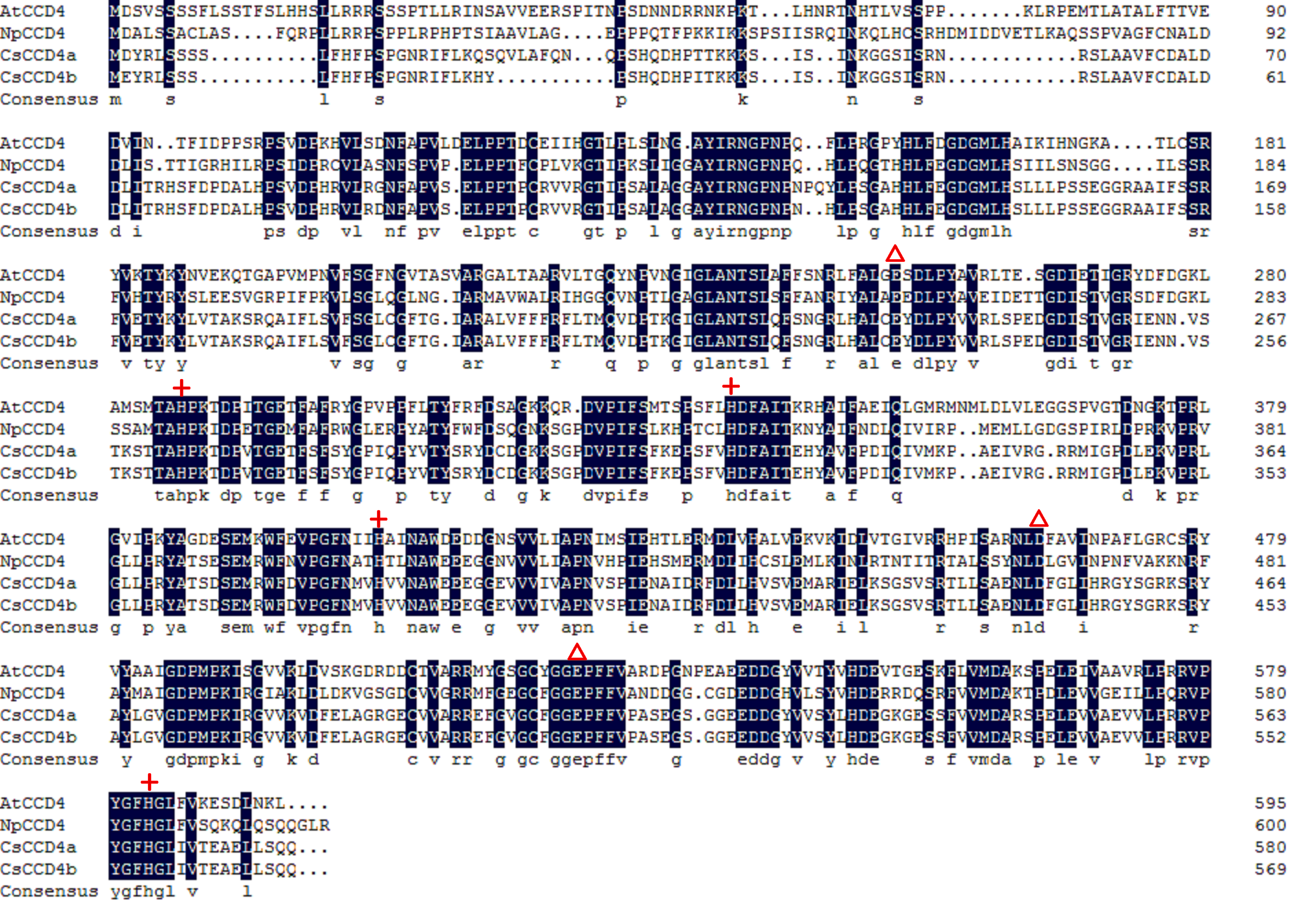

Supplement: Supplementary file 1 [file ijms-19-04006-s001.zip › Supplementary Fig. S8.tif]
